# Combined ketone body and glutamine supplementation restores aerobic energy production in AGC1-deficient neuronal progenitors

**DOI:** 10.1038/s41419-025-08314-4

**Published:** 2025-12-15

**Authors:** Simona Nicole Barile, Maria Chiara Magnifico, Eleonora Poeta, Felix Distelmaier, Luigi Viggiano, Nicola Balboni, Michele Protti, Sabrina Petralla, Antonella Pignataro, Giacomo Volpe, Monica De Luise, Massimo Bonora, Marica Antonicelli, Giorgia Babini, Francesca Massenzio, Vito Porcelli, Eleonora Lama, Roberto Arrigoni, Isabella Pisano, Veronica Addabbo, Anna Campana, Francesca Begnozzi, Stewart A. Anderson, Giuseppe Fiermonte, Federico Manuel Giorgi, Paolo Pinton, Ferdinando Palmieri, Giuseppe Gasparre, Laura Mercolini, Luigi Palmieri, Douglas C. Wallace, Julia Hentschel, Barbara Monti, Francesco Massimo Lasorsa

**Affiliations:** 1 CNR Institute of Biomembranes, Bioenergetics and Molecular Biotechnologies (IBIOM), Bari, Italy; 2 Department of Biosciences Biotechnologies and Environment, University of Bari “A. Moro”, Bari, Italy; 3 Department of Pharmacy and BioTechnology (FaBiT), Alma Mater Studiorum - University of Bologna, Bologna, Italy; 4 IRCCS Istituto delle Scienze Neurologiche di Bologna, Bologna, Italy; 5 Department of General Pediatrics, Neonatology and Pediatric Cardiology, University Children’s Hospital, Heinrich-Heine University, Düsseldorf, Germany; 6 Hematology and Cell Therapy Unit, IRCCS Istituto Tumori “Giovanni Paolo II”, Bari, Italy; 7 Department of Medical and Surgical Sciences (DIMEC), Alma Mater Studiorum - University of Bologna, Bologna, Italy; 8 Department of Medical Sciences, University of Ferrara, Ferrara, Italy; 9 Department of Psychiatry, The Children’s Hospital of Philadelphia and the University of Pennsylvania, Philadelphia, PA USA; 10 Biomedical Research Center, Kansai Medical University, Hirakata, Osaka Japan; 11 Center for Mitochon drial and Epigeno mic Medicine, Division of Human Genetics, Department of Pediatrics, The Children’s Hospital of Philadelphia, Perelman School of Medicine, Philadelphia University of Pennsylvania, Pennsylvania, PA USA; 12 Institute of Human Genetics, University Hospital Leipzig, Leipzig, Germany

**Keywords:** Mitochondrial proteins, Encephalopathy

## Abstract

AGC1 deficiency is a rare, early-onset encephalopathy caused by mutations in the *SLC25A12* gene, encoding the mitochondrial aspartate/glutamate carrier isoform 1 (AGC1). Patients exhibit epileptic encephalopathy, cerebral hypomyelination, severe hypotonia, and global developmental delay. A hallmark biochemical feature of AGC1 deficiency is reduced brain N-acetylaspartate (NAA), a key metabolite involved in myelin lipid synthesis. However, the underlying mechanisms leading to the hypomyelinating phenotype remain unclear. In this study, we generated neuronal progenitors (NPs) derived from human-induced pluripotent stem cells (hiPSCs) of AGC1-deficient patients to investigate the metabolic and bioenergetic consequences of AGC1 loss. We demonstrated that AGC1-deficient NPs exhibit impaired proliferation, increased apoptosis, and a metabolic shift toward a hyperglycolytic phenotype due to defective mitochondrial pyruvate oxidation. RNA sequencing revealed downregulation of mitochondrial pyruvate carrier MPC1/2, limiting pyruvate-driven oxidative phosphorylation (OXPHOS) and reinforcing glycolysis as the primary energy source. Despite this metabolic shift, AGC1-deficient mitochondria retained the potential for OXPHOS when alternative anaplerotic substrates were provided. Notably, the administration of ketone bodies, in combination with glutamine, fully restored mitochondrial respiration, suggesting a mechanistic basis for the clinical improvements observed in AGC1-deficient patients undergoing ketogenic diet therapy. Our study highlights the importance of alternative metabolic pathways in maintaining neuronal energy homeostasis in AGC1 deficiency and offers insights into potential therapeutic strategies aimed at bypassing the mitochondrial pyruvate oxidation defect.

## Introduction

AGC1 deficiency is a rare early onset encephalopathy caused by mutations in the *SLC25A12* gene encoding the isoform 1 of the mitochondrial aspartate/glutamate carrier [1, 2]. Affected infants develop epilepsy, cerebral hypomyelination, global developmental disability and severe hypotonia as well as additional clinical features that overlap with other oxidative phosphorylation (OXPHOS) disorders [3, 4]. Characteristic biochemical hallmarks include elevated lactate levels in blood and cerebrospinal fluid, and decreased levels of brain N-acetylaspartate (NAA), a precursor of myelin lipids [5]. Low NAA is associated with a hypomyelination, although whether this results from diminished NAA synthesis, defective neuronal development or neurodegeneration remains debated [6].

In mitochondria, AGC1 and its isoform AGC2, encoded by the *SLC25A13* gene, mediate the Ca^2^^+^-regulated exchange of mitochondrial aspartate for cytosolic glutamate/H⁺ across the inner mitochondrial membrane [7]. AGC1 is active in excitable tissues, such as the muscle, heart and brain, whereas AGC2 is mainly expressed in the liver [8, 9]. Under aerobic conditions, AGC activity is crucial for the malate-aspartate shuttle (MAS), a unidirectional route for the re-oxidation of glycolytic NADH reducing equivalents into the mitochondrial respiratory chain (MRC). MAS, the major redox shuttle system in the brain [10], involves the concerted activity of mitochondrial and cytosolic isoforms of both glutamate-oxaloacetate transaminase (GOT) and malic dehydrogenase (MDH), along with the mitochondrial oxoglutarate/malate carrier (OGC, encoded by the *SLC25A11* gene) and AGC, the rate-limiting step of the pathway [11, 12].

In neurons, up to 80% of brain glucose is oxidized to sustain cell proliferation, synapse formation, and myelination during postnatal brain development, and MAS activity mainly relies on AGC1, as AGC2 is poorly expressed [13–15]. In neurons from AGC1-knock-out mice, glucose respiration is severely compromised, forcing glycolytic pyruvate to lactate conversion by lactic dehydrogenase (LDH) and impairing the astrocyte–neuron lactate shuttle (ANLS) unable to sustain neuron energy metabolism [16–18]. Furthermore, AGC1-null mice recapitulate the clinical manifestations of AGC1 deficiency [15, 19], showing brain atrophy, neurodevelopmental delay, seizures, hypomyelination, reduced brain NAA and aspartate content, and early post-natal death. Another AGC1-deleted mouse line failed to produce viable AGC1^−/−^ offspring [20], further highlighting a key role of AGC1 in brain development. Notably, also neural progenitor cells (NPCs) are affected by metabolic alterations, reducing proliferation and neurogenesis. This disruption contributes to abnormal brain development, as observed in other neurodevelopmental disorders [21].

In AGC1 deficiency patients, ketogenic diet (KD) has proven effective in limiting epilepsy and improving muscle tone, and, in some cases, in increasing myelination and attenuating brain atrophy [4, 22]. Although data remain scarce (14 cases from 12 families) [4], these responses suggest a targeted effect of KD on altered brain metabolism in AGC1 deficiency through yet undefined biochemical mechanisms. KD can sustain NPC proliferation and developmental gene expression via epigenetic mechanisms [23], but its therapeutic potential in AGC1-deficient brain cells needs proper validation.

In this study, we modeled AGC1 deficiency by generating human neuronal progenitors (NPs) derived from hiPSCs of two patients carrying distinct AGC1 mutations. These NPs, mimicking early postnatal neurogenesis, displayed proliferation defects and increased apoptosis. Transcriptomic profiling revealed a metabolic reprogramming toward glycolysis due to impaired mitochondrial glucose oxidation. Importantly, administration of ketone bodies (KB) and glutamine, bypassing the identified downregulation of the mitochondrial pyruvate transporter MPC1/2, restored mitochondrial respiration, unveiling a biochemical basis for KD-related clinical improvements.

## Results

### hiPSC-derived NPs from AGC1-deficient patients display impaired proliferation

**Fig. 1:**
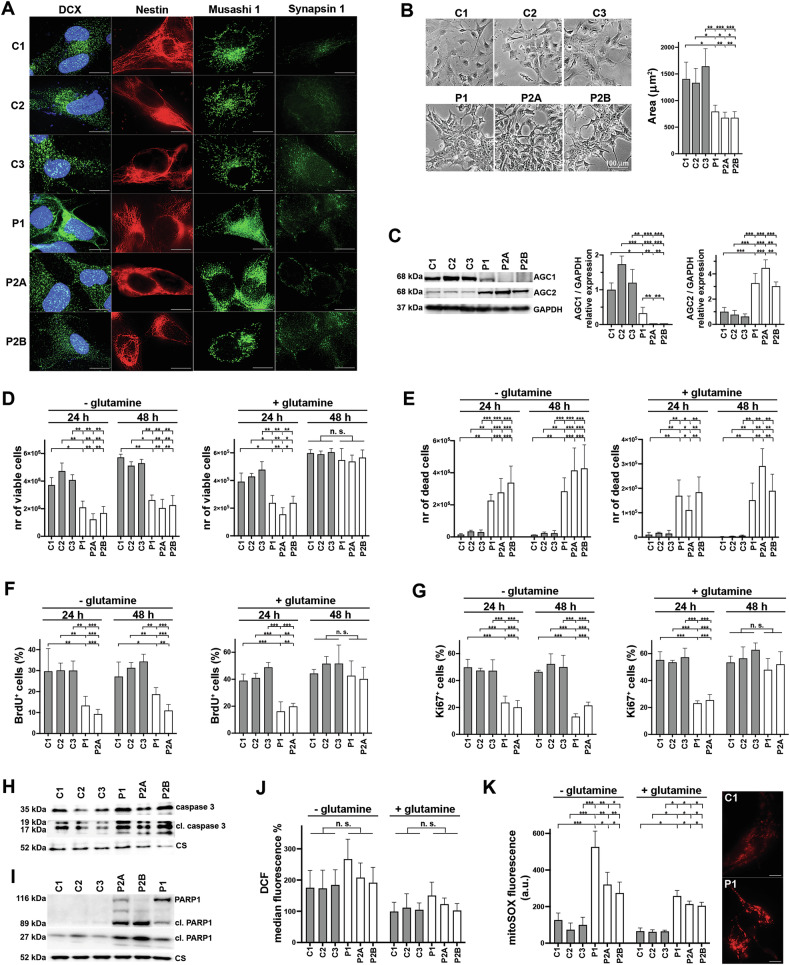
Glutamine dependent proliferation of NPs from hiPSCs of patients with AGC1 deficiency. **A** Characterization of control and patient NPs by Immunofluorescence. NPs were cultured on matrigel-coated 24 mm coverslips and fixed with 4% PFA. Cells were then permeabilized with PBS-0.1% Triton X-100 and subjected to immunostaining with doublecortin (DCX, marker of neural stem cells, NSCs, committed to become neurons), Nestin (marker of NSCs), Musashi 1 (marker of neural precursor cells) and Synapsin 1 (marker of differentiated neurons) antibodies and nuclear staining with DAPI (DCX panels). Scale bars: 10 μm. **B** Representative bright-field images and quantification of total cell area of control (C1, C2, C3; gray bars) and patient NPs (P1, P2A, P2B; white bars). For each experiment, the areas of at least 10 cells were measured using NIS Elements AR software (Fiji, NIH, USA) in each of three randomly selected fields acquired with a 10× objective on an Eclipse TS100 microscope (Nikon, Japan). Data represent the means ± SD of three independent acquisitions. **C** Western blot (WB) analysis of 100 μg of protein of control (C1, C2, C3; gray bars) and patient NPs (P1, P2A, P2B; white bars), probed with antibodies against AGC1, AGC2, and GAPDH. Histogram graphs show the means ± SD of the densitometric ratios of AGC1 or AGC2 to GAPDH from three independent experiments. **D**–**G** Control (C1, C2 and C3, gray bars) and patient NPs (P1, P2A, P2B; white bars) were seeded at a density of 20.000 cells/cm^2^ and grown for 24 and 48 h in DM, with or without 2 mM glutamine. Histograms show the means ± SD of the number of viable (**D**) and nonviable Trypan blue-stained cells (**E**) from three independent experiments. NPs were incubated with 1 μg/mL DAPI and immunostained for the proliferation markers BrdU (**F**) and Ki67 (**G**). Confocal images were acquired using a 60× objective, and the means ± SD of the percentage of BrdU^+^ or Ki67^+^/ total cells were calculated from three independent experiments. **H**, **I** Activation of apoptosis in AGC1 deficient NPs. Representative WB showing the total and cleaved forms of Caspase 3 (**H**) and PARP1 (**I**) in 100 μg of control and patient NPs probed with antibodies against Caspase 3, PARP1 and citrate synthase CS. ROS production was measured in control (C1, C2, C3; gray bars) and patient NPs (P1, P2A and P2B, white bars) incubated 8 h in DM ± 2 mM glutamine and loaded with 20 μM CM-H2DCFDA (DCF, **J**) or with 5 μM MitoSOXred (**K**). DCF fluorescence was measured using cytofluorimetry, while MitoSOXred fluorescence intensity was quantified via fluorescence microscopy in at least 30 cells in each experiment. Data represent the means ± SD of three independent experiments. The images shown are representative of MitoSOXred fluorescence from control C1 and patient P1 NPs incubated in DM without glutamine. Scale bars: 10 μm. *p < 0.05, **p < 0.01 ***p < 0.001 by one-way ANOVA with Tukey’s comparison test.

To dissect the biochemical mechanisms of AGC1 deficiency in human cells, we generated human-induced pluripotent stem cells (hiPSCs) from two individuals with different pathogenic mutations in *SLC25A12*. The first patient (P1) previously reported in [2] harbors a homozygous missense mutation (R353Q) resulting in a variant retaining ~15% of wild-type activity [2]. The second patient (P2) diagnosed by trio-exome sequencing is compound heterozygous for c.225del; p.(Glu76Serfs*17) and c.1747C > A; p.(=) in *SLC25A12* [4]. P2, a male born to healthy non-consanguineous parents, manifested profound developmental delay, microcephaly (−3 SD), and drug-resistant epilepsy starting from 7 months of age. Brain MRI, including MR-spectroscopy, showed elevated myoinositol and reduced NAA peaks (Fig. S1A, B). The synonymous variant in P2 was predicted to alter mRNA splicing (Fig. S1C). RT-PCR analysis performed on patient’s cDNA confirmed skipping of exon 17 (Fig. S1D–G), leading to complete loss of AGC1 protein in both muscle and fibroblasts (Fig. S1H, I and Supplementary Materials 1). P2 fibroblasts displayed normal proliferation, lactate production, mitochondrial mass and membrane potential (not shown), as well as mitochondrial oxygen consumption (Fig. S1J–M). To develop an accurate human in vitro model of AGC1 deficiency, we differentiated hiPSCs of both P1 and P2 into neuronal progenitors (NPs) committed to immature, still proliferating neurons mimicking the early stages of neurogenesis [24] (Fig. 1A). hiPSCs from P1 (one line) and P2 (two clones, P2A and P2B), as well as from three healthy controls (C1, C2 and C3) were differentiated using a modified protocol from Choi’s [25] without vitamin A. Immunofluorescence analysis of NPs from both patients and controls was positive for Nestin, Doublecortin (DCX,) and Musashi-1 (markers of neural precursor cells) and only weakly for Synapsin (marker of differentiated neurons) (Fig.1A), [24, 26]. Conversely, the negative staining of markers for glial precursor cell (NG2) [27], astroglial (GFAP) [28] and oligodendrocyte precursor cell (OLIG2) [29] excluded a non-neuronal differentiation fate (Fig. S2). All patient NPs exhibited diminished cell size and higher cellular aggregation compared with controls (Fig. 1B). WB analysis demonstrated a significant reduction of the mutant AGC1 in P1 and confirmed its absence in both P2 clones (Fig. 1C and Supplementary Material 2).

Since the downregulation of AGC1 inhibits the growth in models of neurons [30] and cancer cells [31], we assayed NPs proliferation. After 24 h in differentiation medium (DM) ± glutamine, all patient NPs exhibited a significant deficit in proliferation versus controls, with recovery at 48 h only in glutamine-supplemented medium (Fig. 1D). Consistently, BrdU incorporation (Fig. 1F) and the Ki67 staining were reduced (Fig. 1G). Notably, all AGC1-deficient NP cultures showed high cell mortality at 24 and 48 h (Fig. 1E), suggesting that cell death mechanisms may concurrently undermine the survival of patient NPs. Indeed, active Caspase 3 and PARP1 cleavage (Fig. 1H, I and Supplementary Material 3) were prominently detected only in patient NPs, indicating an occurring apoptotic process. Analysis of reactive oxygen species (ROS) using dichlorofluorescein (DCF) showed no significant difference between patient and control NPs (Fig. 1J). In contrast, MitoSOX staining revealed higher mitochondrial superoxide levels in patient NPs, which were further exacerbated under glutamine deprivation (Fig. 1K), suggesting that AGC1-deficient NPs are prone to deleterious mitochondrial oxidative stress, particularly when substrate supply to the Krebs cycle is limited.

To understand whether inactive AGC1 affects differentiation into mature neurons, we cultured iPSCs-derived neurospheres in Neurobasal medium to generate cortical glutamatergic neurons [32]. At day 20, when neuronal maturation was addressing to completion, neurite outgrowth and early neuronal networks formation appeared unaffected in patient neurospheres, with no differences in cell morphology and cell-cell interactions compared with controls (Fig. S3A). Furthermore, fluorescence microscopy showed that both control and patient cells were positive for neuronal markers TUJ1, VGLUT and Synapsin, while the glial markers GFAP and OLIG2 were undetectable (Fig. S3B–E). Overall, neurospheres lacking AGC1 may retain their competence to generate mature neurons, although the reduced NP proliferation could result in a lower number of differentiated neurons. Whether mature AGC1-deficient neurons maintain bioenergetic competence, or instead undergo degeneration as previously proposed [6], remains to be established.

### AGC1-deficient NPs exhibit enhanced glycolysis vs impaired glucose and pyruvate oxidation

**Fig. 2:**
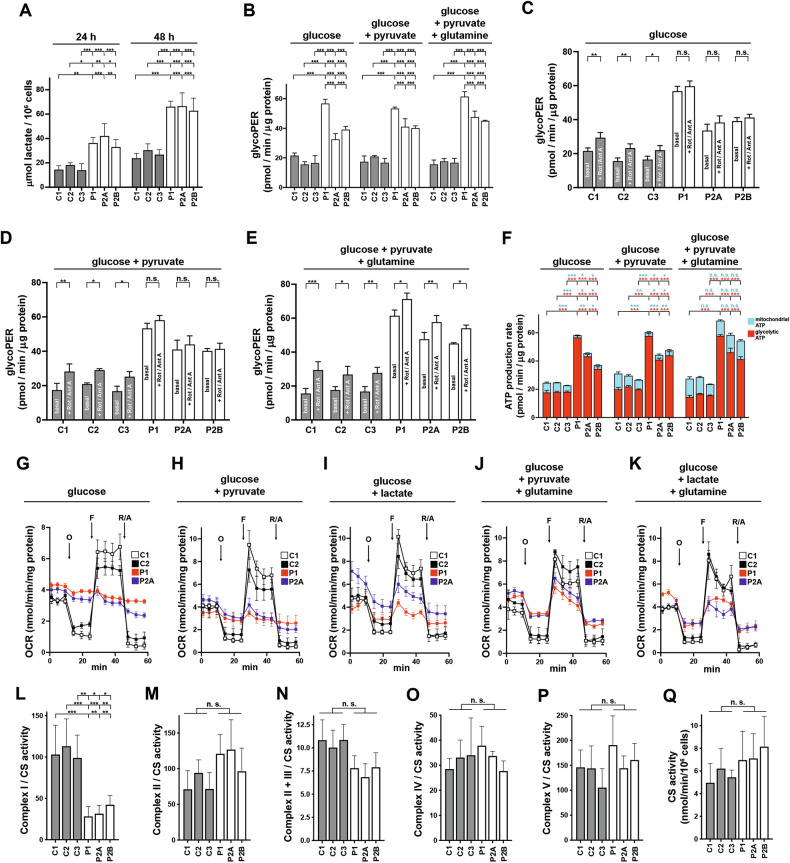
Bioenergetic parameters of NPs with AGC1 deficiency. **A** Lactic acid was quantified in conditioned DM from control (C1, C2, and C3; gray bars) and patient NPs (P1, P2A, and P2B; white bars) harvested 24 or 48 h after the seeding. Values are the means ± SD from three independent experiments performed in triplicate. **B**–**E** The Glycolytic Proton Efflux Rate (glycoPER) was measured in control (C1, C2, and C3; gray bars) and patient NPs (P1, P2A, and P2B; white bars) incubated for 2 h in XF DMEM medium supplemented with 1 g/L glucose, 1 g/L glucose + 1 mM pyruvate, or 1 g/L glucose + 1 mM pyruvate + 2 mM glutamine, and then exposed to the sequential addition of 0.5 μM rotenone + 0.5 μM antimycin A, followed by 50 mM 2-deoxy-D-glucose. Basal glycolysis (**B**) and compensatory glycolysis after addition of rotenone + antimycin under the indicated experimental conditions are compared in (**C**–**E**). Data represent the means ± SD of at least three independent experiments, each including 5–6 replicates per cell type. **F** Real-time ATP rate assays were performed in control and patient NPs incubated for 2 h in XF DMEM Medium supplemented with 1 g/L glucose, 1 g/L glucose + 1 mM pyruvate, or 1 g/L glucose + 1 mM pyruvate + 2 mM glutamine, and then treated with 1.5 μM oligomycin followed by 0.5 μM rotenone/0.5 μM antimycin A. Glycolytic and mitochondrial ATP production rates are shown as red and cyan columns, respectively, with corresponding asterisks marking significant differences. Data represent the means ± SD of at least three independent experiments, each including 5–6 replicates per cell type. Representative mito stress experiments for control C1 (white squares), C2 (black squares), and patient P1 (red squares), and P2A (blue squares) NPs incubated for 2 h in XF base medium supplemented with **G** 1 g/L glucose, **H** 1 g/L glucose + 1 mM pyruvate, **I** 1 g/L glucose + 5 mM lactate, **J** 1 g/L glucose + 1 mM pyruvate + 2 mM glutamine, or **K** 1 g/L glucose + 5 mM lactate + 2 mM glutamine. Cells were then exposed to sequential additions (arrows) of 2 μM oligomycin O, 0.5 μM FCCP F, and 1 μM rotenone + 1 μM antimycin A R/A. Each data point represents the mean ± SD of 4–5 replicates per cell type. The activity of mitochondrial respiratory chain complexes I (**L**), II (**M**), II + III (**N**), IV (**O**), and V (**P**) was determined by spectrophotometry and related to citrate synthase (CS) activity (**Q**) in permeabilized controls (C1, C2, and C3; gray bars) and patients NPs (P1, P2A, and P2B; white bars). Data are the means ± SD of at least three independent experiments. For all the panels shown, a two-way ANOVA analysis with Tukey’s comparison test was performed, except for **C**–**E**, **L**–**P** where one-way ANOVA with Tukey’s test was used; *p < 0.05, **p < 0.01, ***p < 0.001.

Given the role of MAS in aerobic glucose oxidation, we initially assessed the transfer of cytosolic NADH to the mitochondria in patient NPs by measuring lactate release (Fig. 2A). After 24 and 48 h, patient NPs produced roughly twice as much lactate as controls, indicating a glycolytic shift in AGC1-deficient NPs, with NAD⁺ mainly regenerated by LDH. Accordingly, the proton efflux rate (PER) as an indicator of glycolysis activity was markedly enhanced. Basal glycolysis in patient NPs incubated with glucose, glucose + pyruvate, or glucose + pyruvate and glutamine was significantly higher than in controls (Fig. 2B). Moreover, patient NPs exposed to glucose or glucose plus pyruvate (Fig. 2C, D) failed to increase glycolysis upon OXPHOS inhibition with rotenone and antimycin A, whereas glutamine supplementation induced a slight but significant compensatory glycolytic response (Fig. 2E). Notably, total ATP synthesis in patient NPs was markedly higher than in controls (Fig. 2F). Specifically, AGC1-deficient NPs incubated with glucose or glucose plus pyruvate showed the expected higher glycolysis-mediated ATP production alongside a reduced mitochondrial synthesis. However, glutamine addition also enhanced mitochondrial ATP production, which was no longer significantly different from that of control NPs.

To define the oxidative properties of the investigated NPs, we measured their oxygen consumption rates (OCRs) [33]. Patient NPs were highly inefficient in oxidizing glucose alone or in combination with pyruvate or lactate (Fig. 2G–I), since basal mitochondrial respiration, ATP-linked OCR, maximal respiration and spare respiratory capacity were significantly reduced (Fig. S4A–C). A partial rescue of mitochondrial respiration was achieved with glutamine supplementation, particularly basal and ATP-linked OCR (Figs. 2J, K and S4D, E), although maximal respiration and spare capacities remained low, revealing that mitochondria of AGC1-deficient NPs operate near their bioenergetic limit (Figs. 2J, K and S4D, E). Increased proton leak/mitochondrial basal respiration ratios (Fig. S4A–E) further demonstrated inefficient coupling between substrates oxidation and ATP synthesis, whereas the pronounced non-mitochondrial respiration measured in mutant cells (Fig. S4A–E) suggested activation of alternative oxygen-consuming mechanisms insensitive to mitochondrial inhibitors. Notably, these mitochondrial respiration defects were absent in the corresponding hiPSCs (Fig. S5A, B). We also assessed whether MRC complexes were affected in AGC1-deficient cells. Complex I (CI) resulted the only complex with significantly reduced activity in all patient NPs (Fig. 2L), potentially limiting mitochondrial NADH reoxidation and favoring glycolysis, whereas Complexes II, II + III, IV, and V were unaffected (Fig. 2M–P). All MRC activities were normalized to the TCA cycle enzyme citrate synthase (CS), which showed similar expression and activity across all NPs (Fig. 2Q). These results indicate that patient NPs rely on upregulated glycolysis to meet energy demands due to limited pyruvate oxidation, while retaining OXPHOS capacity when provided with anaplerotic substrates such as glutamine.

### Mitochondrial biogenesis is increased in AGC1-deficient NPs

**Fig. 3:**
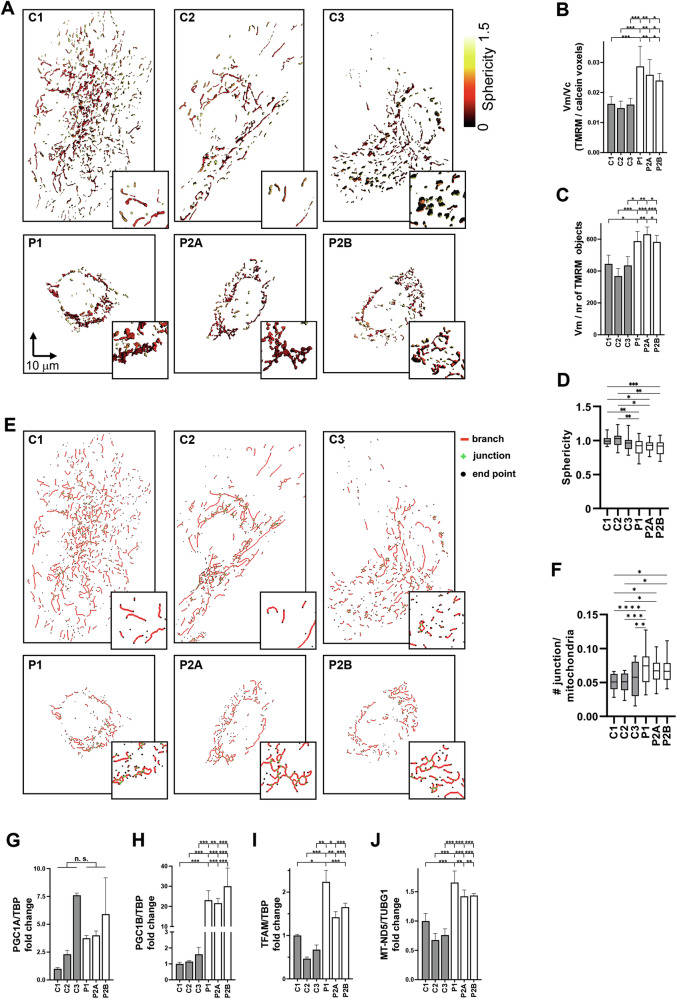
Impact of AGC1 deficiency on mitochondrial network architecture and biogenesis. Control (C1, C2, and C3) and patient NPs (P1, P2A, and P2B) were stained with 1 µM Calcein-AM to assess cellular volumes and with 2 nM ΔΨm-driven TMRM. Z-stack acquisitions for Calcein-AM and TMRM fluorescence were deconvolved using Huygens Essential software using theoretical PSF (see Fig. S6). **A** Representative surface rendering of mitochondrial network from control (C1, C2, and C3) and patient NPs (P1, P2A, and P2B). False colors represent mitochondrial sphericity index. **B**, **C** Quantification of mitochondrial density expressed as total mitochondrial volume (Vm, TMRM voxels) divided by cellular volume (Vc, calcein voxels) and **C** average mitochondrial volume (expressed as Vm divided by the number of mitochondria) in control (C1, C2, and C3; gray bars) and patient NPs (P1, P2A, and P2B; white bars). **D** Quantification of mitochondrial sphericity index, calculated as the average sphericity of all mitochondria per cell, in patient (P1, P2A, and P2B; white bars) versus control (C1, C2, and C3; gray bars) NPs. **E** Representative images of mitochondrial arborization in control and AGC1-deficient NPs, obtained by skeletonizing TMRM fluorescence images using ImageJ. **F** Mitochondrial network complexity, assessed by quantifying the number of junctions per mitochondrion, in control (gray bars) and AGC1-deficient (white bars) NPs. Expression of mitochondrial biogenesis markers Peroxisome proliferator-activated receptor gamma coactivator 1A PGC1A (**G**), and 1B PGC1B (**H**), TFAM (**I**), and mitochondrial MT-ND5 (**J**), analyzed by qRT-PCR in control (gray bars) and AGC1-deficient (white bars) NPs. Data are expressed using the comparative 2^−ΔΔCt^ method. ΔCt values for PGC1A, PGC1B, and TFAM were normalized to the housekeeping gene TBP, while MT-ND5 levels were normalized to nuclear DNA using the TUBG1 reference gene. Data are presented as means ± SD of at least three independent measurements. *p < 0.05, **p < 0.01, ***p < 0.001, from one-way ANOVA with Tukey’s test.

To assess whether impaired glucose oxidation was associated with changes in mitochondrial structure, we analyzed patient and control NPs by 3D fluorescence microscopy. Patient NPs exhibited reduced cellular and mitochondrial volumes and fewer individual mitochondria (Figs. 3A and S6A–D). Consequently, when mitochondrial volume was normalized to cell volume, mitochondrial density in patient NPs was higher than in controls (Fig. 3B). Flow cytometry with MitoTracker Green confirmed that AGC1-deficient NPs had comparable mitochondrial mass to control cells despite their smaller size (Fig. S6E, F). Moreover, mitochondria in patient NPs displayed larger individual volume, lower sphericity, and an increased number of network junctions (Fig. 3C–F), indicative of reduced fragmentation and enhanced arborization [34]. Notably, ΔΨm in AGC1-deficient NPs was higher than in controls when measured in complete DM (Fig. S6G) i.e., in the presence of respiratory substrates beyond glucose and pyruvate, indicating preserved mitochondrial function. We excluded the possibility that this increase was due to reversed ATP synthase activity under enhanced glycolysis, as oligomycin produced identical effects on ΔΨm in both control and patient NPs (Fig. S6H).

Analysis of transcript levels of mitochondrial biogenesis regulators in patient NPs showed unchanged *PGC1A* but elevated *PGC1B* expression compared to controls (Fig. 3G, H) [35], suggesting activation of mitochondrial biogenesis. This was further supported by increased levels of the mitochondrial transcription factor *TFAM* (Fig. 3I) and a higher relative mitochondrial DNA (mtDNA) copy number (Fig. 3J).

Taken together, these findings suggest that AGC1-deficient NPs undergo mitochondrial rewiring as a functional adaptation rather than a loss of organelle activity. Despite impaired glucose and pyruvate oxidation, AGC1 deficit does not preclude OXPHOS capacity when alternative substrates are available, supporting a metabolic reprogramming that may ultimately sustain the vital functions of AGC1-deficient NPs [36].

### Shortage of TCA cycle intermediates occurs in AGC1-deficient NPs

**Fig. 4:**
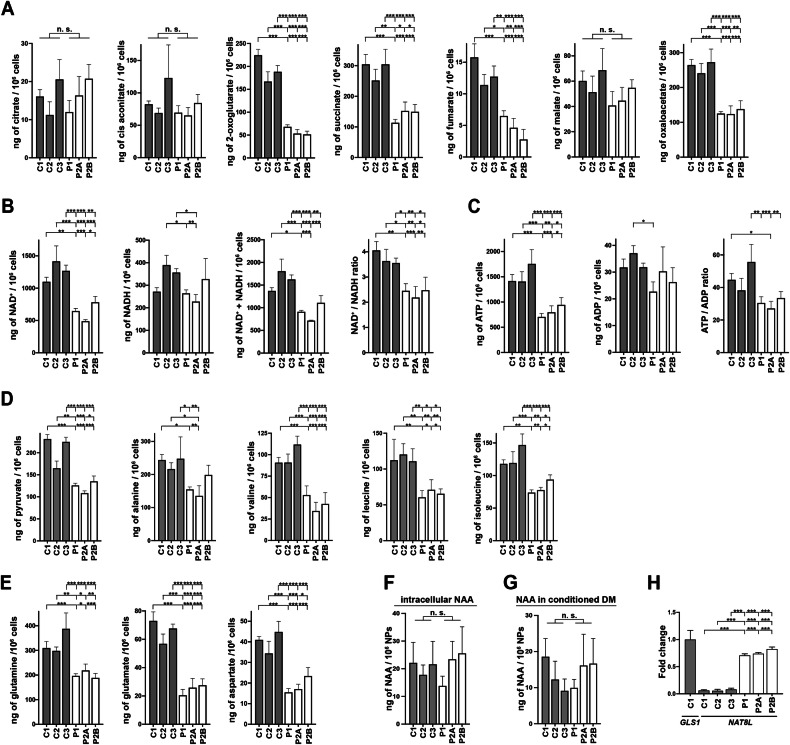
Relative metabolite content in NPs with AGC1 deficiency. Control (C1, C2, and C3; gray bars) and patient NPs (P1, P2A, and P2B; white bars) were harvested after 8 h of incubation with complete DM. The total intracellular pools of **A** TCA cycle metabolites-citrate, cis-aconitate, 2-oxoglutarate, succinate, fumarate, L-malate and oxaloacetate; **B** NAD^+^, NADH, and relative NAD^+^/NADH ratio, **C** ATP, ADP and relative ATP/ADP ratio, **D** pyruvate, alanine, valine, leucine, and isoleucine, **E** the amino acids L-glutamate, L-glutamine and L-aspartate, **F** NAA, and **G** the levels of NAA in conditioned media were determined by HPLC-MS/MS analysis as detailed in “Methods” section. Data are the means ± SD of four independent preparations. **H** Relative expression of the *NAT8L* gene encoding NAA synthase in control (C1, C2, and C3; gray bars) and AGC1-deficient NPs (P1, P2A, and P2B; white bars). Data are expressed using the comparative 2^−ΔΔCt^ method. Given the very low expression of *NAT8L* gene in control NPs, ΔCt values for this gene were normalized to the housekeeping gene *PPIA* and then compared to normalized ΔCt values for the GLS1 gene. Data represent the means ± SD of two independent qRT-PCR experiments. *p < 0.05, **p < 0.01 ***p < 0.001, from one-way ANOVA with Tukey’s test.

Given the impaired mitochondrial activity observed in patient NPs in the presence of pyruvate and glucose, we quantified intracellular metabolites reflecting OXPHOS efficiency [37]. Concerning TCA cycle intermediates, regardless glutamine supplementation, 2-oxoglutarate, succinate, fumarate, and oxaloacetate were markedly decreased in AGC1-deficient NPs, whereas citrate and malate were unchanged. Cis-aconitate was reduced only in patient cells not supplemented with glutamine (Figs. 4A and S7A). This shortage of key TCA cycle metabolites in patient NPs was accompanied by a ~30–50% reduction in the total NAD pool and the NAD⁺/NADH ratio (Figs. 4B and S7B), indicating an imbalanced redox state and altered NAD homeostasis that may affect central energy metabolism and other NAD-dependent cellular pathways [38]. Furthermore, ATP levels were decreased in mutant cells (Figs. 4C and S7C), which, when correlated with the higher ATP production estimated in Fig. 2F, suggests an increased activation of energy-consuming pathways. In NPs with inactive AGC1, we also detected reduced levels of pyruvate and alanine, as well as of valine, leucine, and isoleucine (Figs. 4D and S7D), whose catabolism depends on mitochondrial pyruvate utilization [39]. No changes in the concentrations of these amino acids were instead observed in conditioned media (data not shown). Intracellular glutamine and glutamate levels were decreased compared with controls (Fig. 4E), suggesting higher utilization partially rescuing OXPHOS and promoting cell proliferation [40, 41]. The reduced aspartate may similarly reflect an enhanced utilization to sustain cell viability [42], although it could also result from limited synthesis or export of this amino acid from mitochondria with inefficient AGC1. Notably, both glutamine and aspartate concentrations in culture medium were not significantly different (Fig. S7E).

Our approach cannot conclusively discriminate between altered synthesis or consumption of the investigated molecules. However, our results collectively support a scenario where limited MAS and OXPHOS capacity of patient NPs is compensated by enhanced glycolysis, with an altered balance among several metabolites, that may also exert broader effects on the regulation of cell homeostasis [40].

### Gene expression analysis in AGC1-deficient NPs reveals a profoundly altered transcriptomic profile

**Fig. 5:**
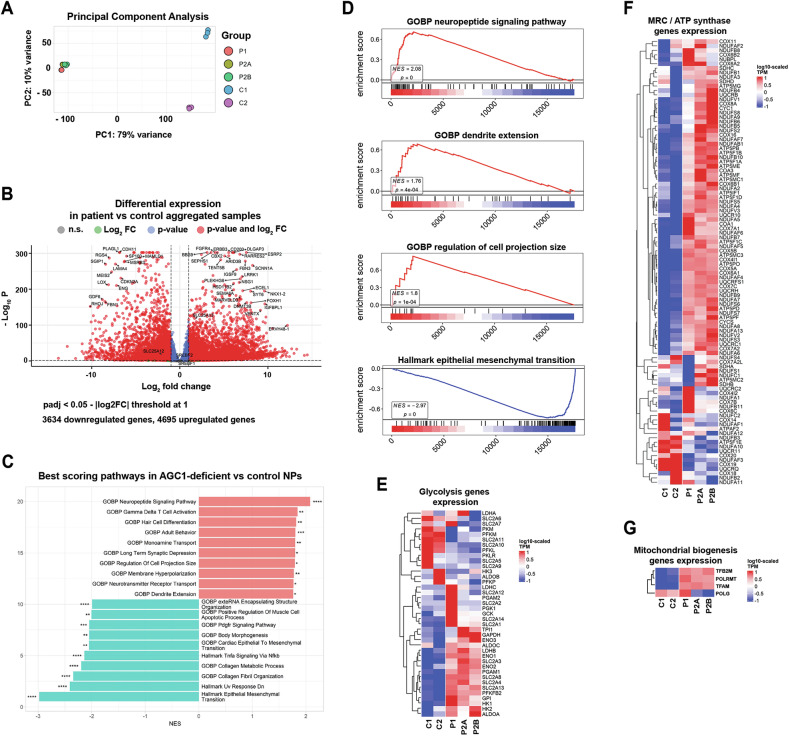
RNAseq analysis revealed a profoundly altered transcriptomic profile in NPs of patients with AGC1 deficiency. **A** Principal component analysis overview of experimental design comparing AGC1-deficient (P1, P2A, P2B) with control (C1, C2) NPs. **B** Volcano plot of differential gene expression in AGC1-deficient NPs vs controls. Thresholds set to p adjusted < 0.05 and |log₂FC|≥1, identifying 3634 downregulated (blue) and 4695 upregulated (red) genes. Dashed lines indicate significance cutoffs. **C** Top scoring pathways identified through gene set enrichment analysis (GSEA) in AGC1-deficient NPs. Significant pathways are ranked by normalized enrichment score (NES), respectively showing the top and bottom 10; *p < 0.05, **p < 0.01, ***p < 0.001, ****p < 0.0001. **D** GO biological process (GOBP) running enrichment score for pathways related to cell surface reshaping and neural differentiation. Heatmaps representing gene expression as log10-scaled TPMs across control (C1, C2) and patient (P1, P2A, and P2B) NPs for **E** glycolysis-related genes, **F** components of MRC/ATP synthase, and **G** genes involved in mitochondrial biogenesis.

**Fig. 6:**
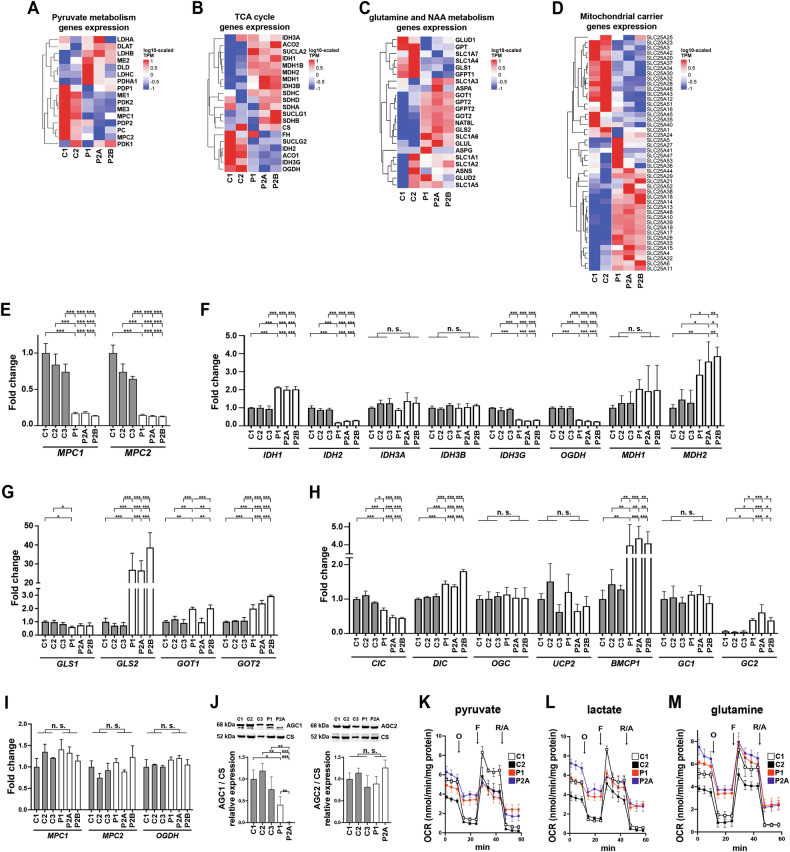
Mitochondrial pyruvate carrier MPC1/2 is downregulated in AGC1 deficient NPs, but not in hiPSCs from patients. Heatmaps representing gene expression as log10-scaled TPMs across control (C1, C2) and patient (P1, P2A, and P2B) NPs for genes related to **A** pyruvate metabolism, **B** TCA cycle, **C** glutamine/glutamate metabolism and NAA synthesis, and **D** for genes of the mitochondrial carrier SLC25 family. qRT-PCR performed in control (C1, C2, and C3; gray bars) and AGC1-deficient (P1, P2A, and P2B; white bars) NPs for **E** *MPC1* and *MPC2* genes, **F** *IDH3A*, *IDH3B*, *IDH3G*, *OGDH* and *MDH2* genes involved in TCA cycle, **G** *GLS1*, *GLS2*, *GOT1* and *GOT2* involved in glutamine metabolism, and **H** *SLC25A1/CIC*, *SLC25A10/DIC*, *SLC25A11/OGC*, *SLC25A8/UCP2*, *SLC25A14/BMCP1*, *SLC25A22/GC1* and *SLC25A18/GC2* genes of the SLC25 family. **I** Relative expression of *MPC1*, *MPC2*, and *OGDH* genes in control (C1, C2, and C3; gray bars) and AGC1-deficient hiPSCs (P1, P2A, and P2B; white bars). In **B**, **F**, the analysis also included *IDH2* encoding the NADP-dependent mitochondrial isocitric dehydrogenase, and *IDH1* and *MDH1* genes encoding the cytosolic isoforms of isocitric and malic dehydrogenases, respectively. Data are expressed using the comparative 2^−ΔΔCt^ method. ΔCt values for the indicated genes were normalized to the housekeeping gene *PPIA*. In **H**, given the very low expression of *SLC25A18/GC2* gene in control NPs, ΔCt values for this gene were normalized to the housekeeping gene *PPIA* and then compared to normalized ΔCt values for the *SLC25A22/GC1* gene. For **E**–**H**, data are the means ± SD of at least three independent qRT-PCR experiments. For **I**, data are the means ± SD of two independent experiments. **J** Western blot and densitometric analysis of 100 mg of control (C1, C2, and C3; gray bars) and AGC1-deficient (P1, P2A; white bars) hiPSCs lysate probed with antisera against the indicated proteins. Representative WBs are shown, while histograms display the means ± SD of the densitometric ratios between AGC1 or AGC2 and citrate synthase (CS) from three independent experiments. **K**–**M** Representative mito stress experiments for control C1 (white squares), C2 (black squares), and patient P1 (red squares), and P2A (blue squares) NPs incubated for 2 h in XF base medium supplemented with **K** 1 mM pyruvate, **L** 5 mM lactate, or **M** 2 mM glutamine. Cells were then exposed to sequential additions (arrows) of 2 μM oligomycin O, 0.5 μM FCCP F, and 1 μM rotenone + 1 μM antimycin A R/A. Each data point represents the mean ± SD of 4–5 replicates per cell type. *p < 0.05, **p < 0.01, ***p < 0.001, from one-way ANOVA with Tukey’s test.

RNA sequencing revealed that all patient samples (P1, P2A, and P2B) clustered together and separately from the two sequenced controls (C1, C2; see principal component analysis PCA, in Fig. 5A), validating the robustness of our cell models and suggesting that different AGC1 mutations similarly impact global gene expression. Differential expression analysis identified 4695 upregulated and 3634 downregulated genes in patient NPs (Fig. 5B). Gene Set Enrichment Analysis (GSEA) of Gene Ontology (GO) and Hallmark pathway databases revealed positive enrichment of neuropeptide signaling, regulation of cell projection size, and dendrite extension, together with downregulation of epithelial–mesenchymal transition (EMT), suggesting that AGC1-deficient cells may be more prone to neural differentiation (Fig. 5C, D). Transcriptomic data also assessed the molecular basis of the biochemical phenotype of AGC1-deficient NPs. Key genes associated with glucose uptake and glycolysis were upregulated, along with most mitochondrial respiratory chain subunits and regulators of mitochondrial biogenesis (Fig. 5E–G). Importantly, both *MPC1* and *MPC2*, encoding the mitochondrial pyruvate carrier [43], were markedly downregulated (Fig. 6A, E), thereby impeding mitochondrial respiration in patient NPs even when lactate or pyruvate were supplied as the sole respiratory substrates (Figs. 6K, L and S8A, B). Conversely, transcript levels of *PDHA1*, *DLD*, and *DLAT* encoding the mitochondrial pyruvate dehydrogenase subunits (Fig. 6A) as well as those of TCA cycle enzymes (Fig. 6B, F) were unchanged. An exception was *OGDH* transcript that was reduced by ~60% in mutant NPs, potentially limiting 2-OG oxidation.

In contrast, *GLS2*, encoding phosphate-activated glutaminase was highly upregulated in patient NPs (Fig. 6C, G). Other key genes involved in glutamine metabolism (*GLUD1*, *GOT1/2*, and *GPT1/2*) were found at comparable or higher levels than in controls, as were the mitochondrial glutamate transporters *SLC25A22/GC1* and *SLC25A18/GC2* (Fig. 6D, H). In line with metabolomic data, these findings indicate sustained glutamine metabolism, consistent with the significant rescue of mitochondrial respiration observed in patient NPs when glutamine was used as the sole respiratory substrate (Figs. 6M and S8C).

AGC1-deficient NPs displayed unaltered or higher transcript levels of MAS components, including *MDH1*/2*, GOT1*/*2,* and *SLC25A11/OGC* (Figs. 6B–D, F–H). Conversely, the second mitochondrial aspartate/glutamate carrier SLC25A13/AGC2, sharing identical catalytic activity with AGC1 [7], was significantly upregulated (Figs. 1C and 6D). These findings suggest that MAS machinery is maintained despite AGC1 loss, although potentially operating in a compromised bioenergetic picture, due to an inadequately fed TCA cycle and reduced NADH dehydrogenase activity. Notably, *MPC1*/*MPC2* and *OGDH* downregulation together with increased AGC2 occurred only in patient NPs, and not in the corresponding iPSCs (Fig. 6I, J and Supplementary Material 4), indicating that compensatory transcriptional changes, may arise from alterations in the metabolic switch from highly glycolytic iPSCs [44] to OXPHOS-dependent NPs.

### Ketone bodies restore cell respiration in AGC1-deficient NPs

**Fig. 7:**
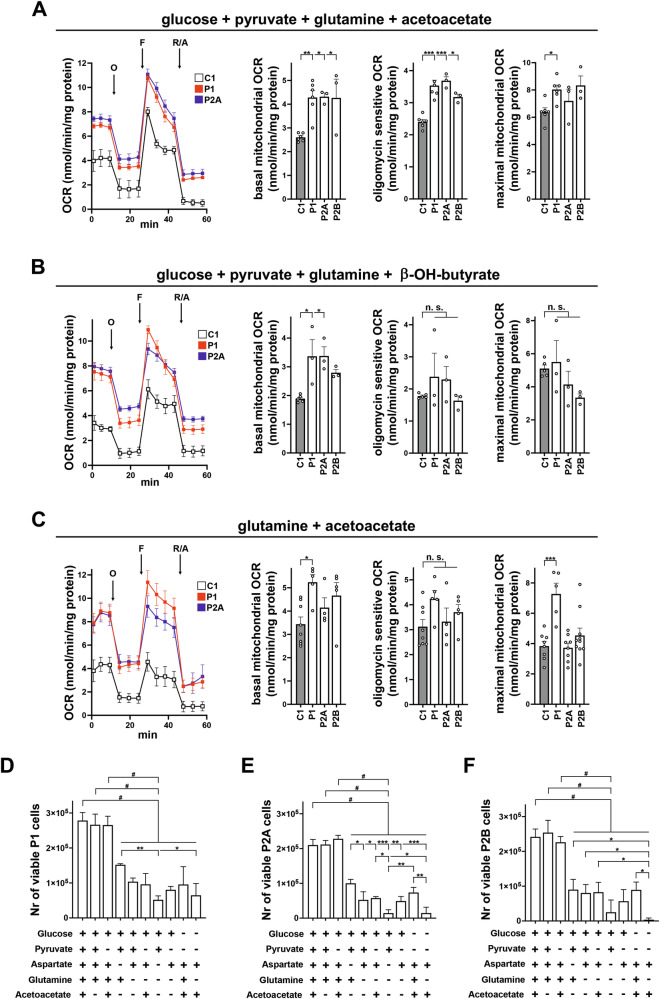
Ketone bodies restore mitochondrial respiration in NPs with AGC1 deficiency. Representative OCR traces and calculated mitochondrial basal respiration, oligomycin sensitive respiration (i.e., ATP turn over), and maximal FCCP sensitive respiration from mito stress experiments performed with control (C1, gray bars) and AGC1-deficient (P1, P2A, and P2B; white bars) NPs incubated for 2 h in XF base medium supplemented with **A** 1 g/L glucose + 1 mM pyruvate + 2 mM glutamine + 5 mM acetoacetate, **B** 1 g/L glucose + 1 mM pyruvate + 2 mM glutamine + 5 mM b-OH-butyrate, or **C** 2 mM glutamine + 5 mM acetoacetate. Cells were then exposed to sequential additions (arrows) of 2 μM oligomycin O, 0.5 μM FCCP F, and 1 μM rotenone + 1 μM antimycin A R/A, and bioenergetic parameters measured as previously described [33]. Data are the means ± SD of at least three independent experiments, each including 4–5 replicates per cell type. Glucose, glutamine and aspartate are indispensable for AGC1 deficient NPs proliferation. Trypan blue exclusion assays were performed with P1 (**D**), P2A (**E**) and P2B (**F**) AGC1 deficient NPs seeded at a density of 20000 cells/cm² and grown 24 h in XF base medium supplemented with 15 mM HEPES, 10 ng/ml bFGF, 10 ng/ml EGF, 1% N2 supplement, 2% Neuro-Brew-21 w/o vitamin A, 50 ng/ml BSA, 0.5 mM Vitamin B12, 14 nM biotin, 15 mM hypoxanthine, 0.5 mM lipoic acid, 1.5 mM thymidine, 49 mM alanine, 50 mM asparagine, 100 mM cysteine, 50 mM glutamate, 150 mM proline. The medium also contained 1 g/L glucose, 1 mM pyruvate, 2 mM glutamine, 5 mM acetoacetate and 50 μM aspartate in the indicated combinations. Data are the means ± SD of at least three independent assays. *p < 0.05, **p < 0.01, ***p < 0.001, ^#^p < 0.0001 by one-way ANOVA with Tukey’s comparison test.

The bioenergetic and transcriptomic features of AGC1-deficient NPs indicate minimal margins to sustain OXPHOS using primary neuronal substrates, such as glucose, lactate and pyruvate. In contrast, glutamine partially alleviated the bioenergetic deficit and supported cell proliferation. KD, which has been reported to ameliorate AGC1 deficiency symptoms [22, 45], promotes β-oxidation, thereby increasing hepatic production of the ketone bodies (KB) acetoacetate and β-hydroxybutyrate, which serve as alternative energy source in brain and muscle. Indeed, in patient NPs, β-hydroxybutyrate and, to a greater extent, acetoacetate produced a robust recovery of mitochondrial respiration parameters when combined with glucose, pyruvate, and glutamine (Figs. 7A, B and S8D, E), except for the non-mitochondrial respiration and proton leak. Interestingly, OCR compensation was also observed when acetoacetate was combined with glutamine in the absence of glucose (Figs. 7C and S8F), that inhibited the effect of KB at low concentrations (Figs. S8G and S9).

To verify whether KB influence cell proliferation, NPs were cultured in minimal media supplemented with growth factors, coenzymes, and amino acids (see “Methods”), along with combinations of glucose, pyruvate, glutamine, aspartate, and acetoacetate (Figs. 7D–F and S10). Control NPs proliferated normally under all conditions, whereas the number of AGC1-deficient NPs remained significantly lower and comparable to that observed in complete media only when glucose, glutamine, and aspartate were present, regardless of acetoacetate or pyruvate addition. Importantly, scarce proliferation occurred in patient NPs grown in media lacking, glucose, glutamine and aspartate, indicating that these substrates remain essential for NPs viability. Overall, KB strongly support mitochondrial respiration but are dispensable for proliferation of AGC1-deficient NPs highlighting a biochemical scenario where mitochondrial function depends on alternative substrates.

### NAA synthesis is unaltered in AGC1-deficient NPs

Reduced NAA levels in the brain are a hallmark of AGC1 deficiency. NAA is synthesized in neurons by the aspartate N-acetyltransferase (ANAT) encoded by the *NAT8L* gene [46], whose expression was low in control NPs but significantly upregulated in mutant NPs (Figs. 4H and 6C). Nevertheless, mass spectrometry revealed comparable NAA levels in patient and control NPs (Fig. 4F) and in their conditioned media (Fig. 4G). These data suggest that in AGC1-deficient NPs, upregulated NAT8L operates in the presence of available substrates for NAA synthesis, namely, aspartate and acetyl-CoA, despite reduced intracellular aspartate (Fig. 4E). Enhanced aspartate utilization may contribute to NAA synthesis, while high glutamine consumption may favor acetyl-CoA generation. In OXPHOS-defective and glycolytic cells, glutaminolysis drives the reductive carboxylation of 2-OG through isocitrate dehydrogenases, producing acetyl-CoA from citrate when OAA is limited [47, 48]. Likewise, *IDH1* upregulation (Fig. 6B, F) encoding cytosolic isocitrate dehydrogenase may sustain cytosolic citrate formation in AGC1-deficient cells, bypassing impaired mitochondria pyruvate entry and limited citrate export to the cytosol, typical of cells with efficient glucose oxidation [11].

### AGC1-deficient NPs exhibit a mutated transcriptomic profile of Fe(II)/2-OG-dependent oxygenase genes involved in chromatin remodeling

**Fig. 8:**
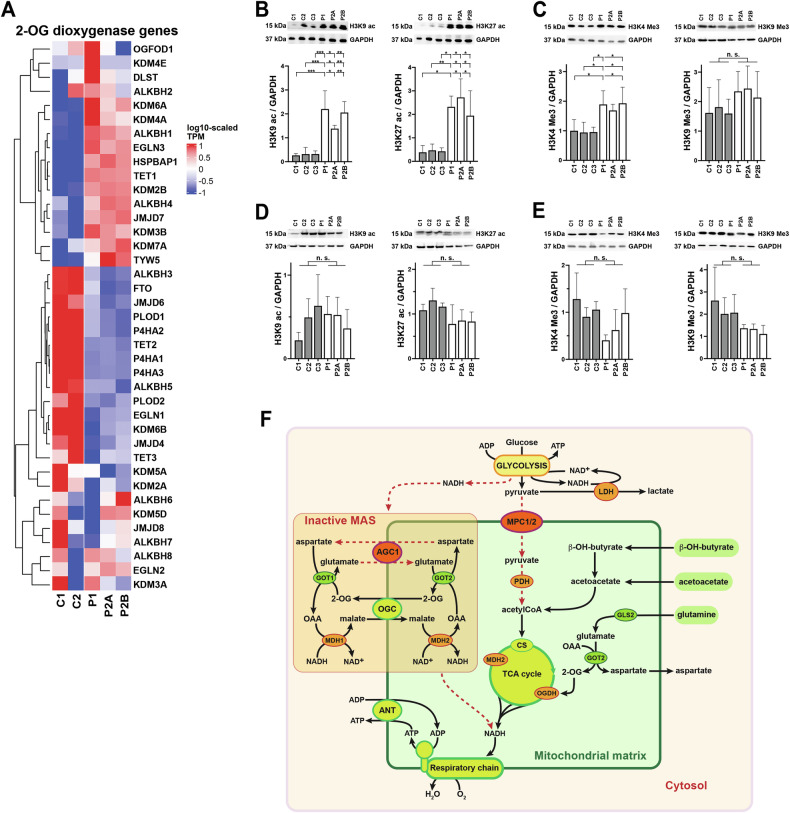
Altered histone post-translational modifications in NPs with AGC1 deficiency. **A** Heatmaps showing gene expression as log10-scaled TPMs across control (C1, C2) and patient (P1, P2A, and P2B) NPs for genes encoding Fe(II)/2OG-dioxygenases. **B**–**E** Representative Western blot images and densitometric analysis of 100 μg of protein from control (C1, C2, and C3; gray bars) and patient (P1, P2A, and P2B; white bars) NPs (**B**, **C**) and hiPSCs (**D**, **E**), probed with antibodies against acetylated lysine 9 (H3K9ac) and 27 (H3K27ac) (**B**, **D**) or trimethylated lysine 4 (H3K4Me3) and 9 (H3K9Me3) (**C**, **E**) of histone H3. Histogram graphs show the means ± SD of densitometric ratios normalized to GAPDH from at least three independent experiments. *p < 0.05, **p < 0.01, ***p < 0.001, by one-way ANOVA with Tukey’s comparison test. **F** In neuronal progenitors from hiPSCs of patients with AGC1 deficiency, the combined supplementation of β-OH-butyrate, acetoacetate and glutamine bypasses impaired mitochondrial pyruvate oxidation, which results from the downregulation of the mitochondrial pyruvate carrier MPC1/2. Dashed red arrows indicate inhibited pathways. MAS malate-aspartate NADH shuttle; AGC1 mitochondrial aspartate/glutamate carrier isoform 1; MPC1/2 mitochondrial pyruvate carrier; OGC mitochondrial oxoglutarate/malate carrier; ANT mitochondrial ATP/ADP carrier; LDH lactic dehydrogenase; PDH pyruvate dehydrogenase; CS citrate synthase; OGDH 2-oxoglutarate dehydrogenase; MDH1 and MDH2, cytosolic and mitochondrial isoforms of malate dehydrogenase; GLS2 glutaminase isoform 2; GOT1 and GOT2 cytosolic and mitochondrial isoforms of glutamate-oxaloacetate aminotransferase.

In AGC1-deficient NPs, glutamine sustained proliferation and, partially, mitochondrial respiration. Accordingly, RNAseq revealed upregulation of glutamine metabolism enzymes (Fig. 6C, G), possibly accounting for the reduced intracellular levels of glutamine, as well as for glutamate and 2-OG (Fig. 4), the major downstream products of glutaminolysis that also fuel several biosynthetic pathways [11, 41, 49]. Moreover, 2-OG and O_2_ are substrates for Fe(II)/2-OG-dependent oxygenases, nuclear enzymes that catalyze oxidative reactions regulating chromatin structure and gene expression [50, 51]. Together with elevated mitochondrial ROS (Fig. 1K), activation of these enzymes could contribute to the high non-mitochondrial OCR observed in AGC1-deficient NPs. We excluded the contribution of NADPH oxidase, as its inhibitor apocynine [52] did not affect OCR (Fig. S5C, D). Transcriptomic data revealed major changes in Fe(II)/2-OG-dioxygenase expression in AGC1-deficient NPs (Fig. 8A), including repression of genes involved in DNA/RNA demethylation (ALKBH3, ALKBH5, FTO, TET2 and TET3) and in histone demethylation (JMJD4 and KDM6B), counterbalanced by upregulation of DNA demethylases TET1, ALKBH1 and ALKBH4, and histone demethylase JMJD7, KDM4A, KDM6A KDM7A, KDM2B, KDM3B. These data point to a modified activation of multiple chromatin regulators. Accordingly, mutant NPs displayed significant H3K9 and H3K27 hyperacetylation and increased H3K4 trimethylation compared with controls, indicative of chromatin activation [53], while H3K9 trimethylation was unchanged (Fig. 8B, C; Supplementary Materials 5 and 6). Notably, control and mutant iPSCs showed no differences in histone acetylation or methylation (Fig. 8D, E; Supplementary Materials 7 and 8). Thus, AGC1-deficient NPs likely possess a distinct chromatin organization, although the specific loci or domains affected remain to be defined.

## Discussion

This study provides the first evidence of how AGC1 insufficiency affects human NPs, a cell type crucial for postnatal neurodevelopment, where metabolic disturbances can critically impair proliferation, leading to defective neurogenesis and neurological dysfunctions [21, 23]. Previous reports from mammalian cell lines [30, 54, 55] or murine models [15, 19, 56, 57] identified AGC1 as a key player in neuronal energy metabolism. The physiological relevance of AGC1 was evidenced in neuron-rich brain areas of murine models [15, 57, 58], where its loss caused growth retardation, seizures, and hypomyelination, mirroring the clinical manifestations of AGC1 deficiency [4]. In tumor cells, AGC1 downregulation impaired mitochondrial respiration and cell proliferation, particularly under glutamine deprivation limiting cytosolic aspartate levels essential for protein and nucleotide biosynthesis in dividing cells [30, 31].

To explore whether similar consequences occur in human brain cells, we generated NPs from iPSCs of two patients carrying distinct pathogenic AGC1 variants. NPs are well suited to evaluate the bioenergetic consequences of AGC1 deficiency in neurons prior to complete differentiation, while retaining proliferative capacity. We found that patient NPs proliferation relied critically on aspartate and glutamine, the latter serving as an aspartate source [59], substantiating a role for AGC1 in proliferation beyond tumor contexts. Upregulation of AGC2 and other SLC25 aspartate carriers, including SLC25A14/BMCP1 (Figs. 1C and 6H) [60], failed to compensate for this defect, suggesting that glutamine-derived aspartate production occurred mainly in the cytosol rather than in mitochondria. Enhanced glutaminolysis in patient NPs is indicated by activation of GLS2 [61, 62] with mitochondrial 2-OG production via GDH and upregulated GOT2. 2-OG enters the TCA cycle, although its oxidation is hindered by downregulated OGDH and reduced CI activity. Inadequate malate oxidation by MDH2 would limit OAA availability for the GOT2 reaction, while malate could be exported from mitochondria via SLC25A10/DIC and SLC25A11/OGC, supporting cytosolic NAD⁺ reduction and OAA formation through MDH1, ultimately producing aspartate via GOT1. Strong downregulation of MPC1/2 confines glycolytic pyruvate to the cytosol where it serves as a preferred acceptor for NADH electrons via the LDH reaction that is thermodynamically more favorable than MDH2 (ΔG°LDH = -25,1 kJ/mol vs ΔG°MDH2 = 297 kJ/mol) [63]. This accounts for high lactate production in patient NPs in the presence of glucose. Conversely, glutamine and KB restore mitochondrial respiration via anaplerosis, particularly in glucose-free media (Fig. 8F), since without cytosolic pyruvate, the MRC becomes the primary, albeit not efficient, acceptor for NADH reoxidation. Under these conditions, malate oxidation and OAA generation via MDH2 can resume, allowing aspartate export via AGC2 or other carriers [60, 64]. However, glucose deprivation abolished patient NPs proliferation, consistent with its role in providing intermediates for cell growth and antioxidant defense [65]. Moreover, upregulation of cytosolic IDH1 indicated enhanced reductive carboxylation of glutamine-derived 2-OG providing citrate and acetylCoA for lipid biosynthesis and proliferation [47].

The strong rescue of mitochondrial respiration by acute administration of KB and glutamine supports the rationale for ketogenic therapy in AGC1 deficiency [4, 22, 45]. In AGC1-null mice, β-OH-butyrate restored respiration with neuroprotective effect [56]. KB, particularly β-OH-butyrate, also serve as signaling molecules and epigenetic modifier by inhibiting histone deacetylases (HDACs), inducing histone hyperacetylation and promoting specific changes in gene expression [66–69]. Indeed, AGC1-deficient NPs display extensive transcriptomic remodeling and altered histone acetylation/methylation profiles, suggesting a shift in the metabolic-epigenetic interplay emerging during NP differentiation, possibly driven by altered nutrient utilization. The unbalanced expression of 2-OG-oxygenases may further influence chromatin activation and dictate a different metabolic fate in patient NPs.

Overall, AGC1 impairment profoundly reshapes the metabolic landscape of human NPs resembling early neuronal lineage proliferating in adult neurogenic regions, such as the subventricular zone and the dentate gyrus of the hippocampus [24, 70, 71]. These areas generate new neurons contributing to brain homeostasis and repair, and their dysregulation is implicated in several neurological disorders [72–74]. The constrained proliferation, high cell death, and defective glucose oxidation observed in this model of AGC1 deficiency imply that patient neuroprogenitors are vulnerable when deprived of complementary substrates. Nevertheless, their response to glutamine and KB reveals adaptive pathways, reactivating OXPHOS and promoting cell growth, plausibly sustaining neurogenesis. These insights provide a metabolic rationale for ketogenic-based therapies in AGC1 deficiency and related neurological syndromes with overlapping metabolic and neurodevelopmental abnormalities.

## Methods

### Cell lines and differentiation of human induced pluripotent stem cell (hiPSC) lines into neuronal progenitors (NPs)

Control and patient fibroblasts were cultured up to #6 passages in DMEM (cat. 21969035, ThermoFisher) supplemented with 2 mM glutamine and 10% FBS (cat. A5256701, ThermoFisher) at 37 °C in 5% CO₂. Control hiPSC lines included fibroblast-derived C1 BYS0112 (ACS-1026™, ATCC), PBMC-derived C2 hiPSCs [75], and fibroblast-derived C3 hiPSCs (cat. IPS17-00012, Stem Cell Technology Center, Radboudumc, the Netherlands). Patient 1 (P1) hiPSCs were reprogrammed from PBMCs of a male with homozygous c.1058G > A mutation in *SLC25A12* [2] using the CytoTune™-iPS 2.0 Sendai Reprogramming Kit (ThermoFisher). Patient 2 (P2A, P2B) hiPSCs were generated at the SCTC (Radboudumc Nijmegen, the Netherlands) by lentiviral reprogramming of fibroblasts from a 2-year-old male carrying compound heterozygous *SLC25A12* variants (c.225del; p.(Glu76Serfs*17) and c.1747 C > A; p.(=)) [4]. All reprogrammed lines displayed stable karyotype, were mycoplasma-free, and expressed pluripotency markers NANOG, OCT4, SSEA3, SSEA4, TRA-1-60 and TRA-1-81 (certificates of analysis for patient hiPSCs are available). hiPSCs were expanded on hESC-Qualified Matrigel (cat. 354277 Corning) at 37 °C in 5% CO₂ in StemMACS iPS-Brew XF medium (cat. 130107086, Miltenyi Biotec) with 10 μM Y-27632 (cat. 72307 STEMCELL Technologies). Neuronal progenitors (NPs) were derived according to Choi et al. [29] with minor modifications. hiPSCs were passaged as single cells and cultured for 3–4 days in ultra-low adhesion plates (cat. 3471, Corning) without Matrigel, in Differentiation Medium (DM) composed of a 1:1 mixture of DMEM/F-12 (cat. 21331020, ThermoFisher Scientific) and Neurobasal Medium (cat. 17502001, ThermoFisher Scientific), supplemented with 1% N2 (cat. 17502001, ThermoFisher Scientific), 2% Neuro-Brew-21 without vitamin A (cat. 130097263, Miltenyi Biotec), 10 ng/ml EGF (cat. 130097750, Miltenyi Biotec), 10 ng/ml FGF-2 IS (cat. 130104922, Miltenyi Biotec), 50 µg/ml bovine serum albumin (cat. A7030, Merck), 2 mM L-glutamine (cat. 25030081, ThermoFisher Scientific), and 10 units/ml penicillin with 10 µg/ml streptomycin (Merck). The resulting embryoid bodies were then plated on Matrigel-coated dishes and differentiated for two weeks in the final DM without Neurobasal. During differentiation, NPs were passaged weekly using Accutase (cat. 07920, STEMCELL Technologies) and maintained up to passage #12. Patient enrollment was approved by informed parental consent.

### Characterization of NPs and neurospheres by immunofluorescence analysis

Fixed cells were permeabilized with PBS-0.1% Triton X-100 and incubated according to the manufacturer’s instructions with the following primary antibodies: rabbit anti-Doublecortin (cat. ab207175, Abcam), rabbit anti-Musashi-1 (cat. ab52865, Abcam), mouse anti-Nestin (cat. MAB1259, R&D System), mouse anti-Synaspsin I (cat. ab254349, Abcam), mouse anti-SSEA-4 (Cat. sc-21704, SantaCruz Biotechnology), rabbit anti-Olig2 (cat. sc-48817, SantaCruz Biotechnology), mouse anti-GFAP (cat. sc-33673, SantaCruz Biotechnology), rabbit anti-NG2 (cat. ab83178, Abcam), rabbit anti-vGLUT antibody (cat. ab72311, Abcam), and mouse anti-Beta-Tubulin III antibody-Clone TUJ1 (cat. 60052, STEMCELL Technologies). Depending on the primary antibody, cells were then incubated with the appropriate fluorescent secondary antibody, i.e., anti-rabbit IgG Alexa Fluor 488 (cat. A11008, ThermoFisher Scientific), anti-mouse IgG Alexa Fluor 488 (cat. A11001, ThermoFisher Scientific), anti-rabbit IgG Alexa Fluor 546® (cat. A11010, ThermoFisher Scientific), and anti-mouse IgG Alexa Fluor 546® (cat. A11003, ThermoFisher Scientific). Nuclei were stained with DAPI (1 μg/mL, cat. D1306, ThermoFisher Scientific), or alternatively, with Hoechst 33258 (2 μg/mL, cat. 94403, Merck). Images were taken with a Plan-Apochromat 63X/1.4 oil objective on an inverted Zeiss Axiovert 200 high-resolution epifluorescence microscope (Carl Zeiss Jena, Germany) equipped with a CoolSNAP HQ CCD (Roper Scientific, USA) using the Metamorph software (Universal Imaging Corporation, USA). Where specified, images were acquired with a Plan-Apochromat 60X/1.49 oil objective on a Nikon EZ-C1 confocal microscope and analyzed through ImageJ2 Fiji software (NIH, USA).

### Assessment of mitochondrial structural and functional parameters

For mitochondrial structure and membrane potential analyses, 5 × 10⁴ NPs were seeded onto Matrigel-coated glass coverslips and co-loaded with 1 µM calcein acetoxymethyl ester (Calcein-AM, cat. C3100MP, ThermoFisher) and 2 nM tetramethylrhodamine methyl ester (TMRM; cat. T668, ThermoFisher) for 30 min at 37 °C and 5% CO_2_. Z-stack acquisitions (51 planes, 0.3 μm) were captured from at least six different fields using a Plan-NeoFluar 63X/1.3 oil objective on a Zeiss Axiovert 200 inverted epifluorescence microscope (Carl Zeiss Jena, Germany), equipped with a CoolSNAP HQ CCD camera (Roper Scientific, USA). Exposure times were set to 200 ms for TMRM (ex/em: 545/595 nm) and 50 ms for Calcein-AM (ex/em: 494/517 nm). Z-stacks were deconvolved using Huygens Essential software (Scientific Volume Imaging B.V., The Netherlands) using a theoretical PSF. After image reconstruction, single cells were isolated from each acquisition and morphological indexes were extracted using two parallel approaches. Cell and mitochondrial volumes were obtained using Imaris 7 software (Bitplane, Switzerland). Per each cell, two isosurfaces were created from the TMRM and Calcein fluorescence channels, thresholds were automatically calculated using the Ridler Calvar algorithm. The TMRM isosurface was used to quantify mitochondrial membrane potential (TMRM average intensity), number of mitochondria (TMRM objects), and total mitochondrial volume (TMRM voxels), while the Calcein isosurface was used to calculate total cellular volume (Calcein voxels). Quantitative data were obtained using Imaris statistics tool. Reconstructed TMRM images were next processed in ImageJ (https://imagej.net/ij/) to calculate mitochondrial sphericity and arborization. Per each cell, TMRM channels were binarized using the Ridler Calvar algorithm, and mitochondrial sphericity was calculated using the 3D suite plugin [76]. Binarized images were skeletonized using the Skeletonize3D plugin [77] to obtain the number of junctions per branch. To assess the mitochondrial mass, 5 × 10^5^ NPs/mL were stained with 100 nM MitoTrackerTMGreen FM (cat. M46750, ThermoFisher) for 1 h at 37 °C, and fluorescence intensity was measured through an AttuneTM NxT Flow Cytometer with NxT software v.2.6. (ThermoFisher) using a 488-nm excitation laser and 530/30-nm emission filter. For total intracellular ROS quantification, 5 × 10^5^ NPs were incubated for 8 h in DM ± 2 mM glutamine, then resuspended and stained with 20 μM 2′,7′-dichlorodihydrofluorescein diacetate (H2DCFDA; cat. D399, ThermoFisher) for 30 min at 37 °C in Krebs-Ringer buffer (KRB: 135 mM NaCl, 5 mM KCl, 1 mM MgSO4, 0.4 mM KH2PO4, 20 mM HEPES, pH 7.4) supplemented with 1 mM CaCl_2_, 1 g/L glucose, 1 mM pyruvate ± 2 mM glutamine. Positive cells were analyzed using Attune NxT software v.2.6. For mitochondrial hydrogen peroxide measurements, NPs seeded on 24 mm glass coverslips were incubated for 8 h in DM ± 2 mM glutamine, then loaded for 30 min with 5 μM MitoSOX Red (cat. M36007 ThermoFisher Scientific) diluted in KRB with 1 mM CaCl_2_, 1 g/L glucose, 1 mM pyruvate ± 2 mM glutamine at 37 °C. Images were acquired with a Plan-NeoFluar 63X/1.3 oil objective on a Zeiss Axiovert 200 inverted epifluorescence microscope equipped with a CoolSNAP HQ CCD camera using the Metamorph software.

### Cell proliferation assays

For cell proliferation assays, cells were seeded at a density not lower than 10,000 cells/cm^2^ to avoid culture loss of patient-derived NPs. For the Trypan blue exclusion assay, NPs were seeded at 20,000 cells/cm² on Matrigel-coated 6-well plates. After growth under the specified experimental conditions, both floating cells from the conditioned medium and those detached with Accutase were collected and mixed with 0.4% (w/v) Trypan blue (cat. T8154, Merck). Viable and nonviable cells were counted using a Bürker chamber under an Eclipse TS100 microscope (Nikon). For BrdU incorporation assays, NPs were plated on 13-mm Matrigel-coated glass coverslips in DM ± 2 mM glutamine and incubated 24–48 h with 10 μM BrdU (cat. B23151, ThermoFisher). Cells were fixed and processed with rabbit anti-Ki67 (cat. ab15580, Abcam) or rat anti-BrdU (cat. ab6326, Abcam), followed by secondary Anti-Rat IgG AlexaFluor 488 (cat. ab150157, Abcam) or anti-Rabbit IgG AlexaFluor 555 (cat. ab150078, Abcam). Nuclei were labeled with 1 μg/mL DAPI. Fluorescence images were acquired using a Plan-Apochromat 60×/1.49 oil objective (Nikon EZ-C1 confocal), and the labeling index (BrdU- or Ki67-positive/total DAPI nuclei) was quantified with the Cell Counter plugin in Fiji/ImageJ2.

### Measurements of oxygen consumption (OCR) and extracellular acidification rates (ECAR)

OCR and ECAR were simultaneously measured with the XF96 extracellular flux analyzer (Agilent Technologies) [30]. Cells were grown overnight in their specific medium and subsequently washed with unbuffered Seahorse XF Base Medium (cat. 102353, Agilent Technologies) without added substrates prior to analysis. For Mitostress experiments [33], cells were incubated for 2 h in a humidified non-CO_2_ incubator at 37 °C in Seahorse XF Base Medium supplemented with the indicated substrates. OCR was monitored under basal conditions and after sequential injections of 1.5 μM oligomycin (ATP synthase inhibitor, cat. O4876, Merck), 0.5 μM of carbonyl cyanide 4-(trifluoromethoxy) phenylhydrazone (FCCP, mitochondrial uncoupler, cat. C2920 Merck), 1 μM rotenone (cat. 557368, Merck) and 1 μM and antimycin A (cat. A8674, Merck) inhibitors of complex I and complex III, respectively. In Glycolytic Rate and Real-Time ATP Rate assays, cells were incubated for 2 h in a non-CO_2_ incubator at 37 °C with Seahorse XF DMEM Medium (cat. 103575, Agilent Technologies) supplemented with 10 mM glucose ± 1 mM pyruvate and with or without 2 mM glutamine. The glycolytic Proton Efflux Rate (glycoPER) was calculated from OCR and ECAR measurements under basal conditions and after sequential injection of 0.5 μM rotenone/0.5 μM antimycin A and 50 mM 2-deoxy-D-glucose (2-DG, cat. D3179, Merck), an inhibitor of glycolysis. ATP production rates associated with glucose conversion to lactate (glycolytic ATP) and with OXPHOS (mitochondrial ATP) were determined from OCR and ECAR measurements before and after treatment with 1.5 μM oligomycin followed by 0.5 μM rotenone/0.5 μM antimycin A. GlycoPER and ATP rates were calculated using Seahorse Wave 2.6.1 Software (Agilent Technologies). For each assay, OCR and ECAR values were normalized to total cellular protein content.

### MEPS-HPLC-MS/MS targeted quantitative metabolic profiling of NPs cultures

Targeted quantitative analysis of metabolites in NP cultures was performed as previously described [37], using high-performance liquid chromatography coupled to tandem mass spectrometry (HPLC-MS/MS) and a semi-automated microextraction by packed sorbent (MEPS) protocol for the absolute quantitation of selected metabolites: citrate, aconitate, 2-oxoglutarate, succinate, fumarate, malate, oxaloacetate, NADH, NAD^+^, ATP, ADP, N-acetylaspartate, glutamine, glutamate, aspartate, pyruvate, alanine, valine, leucine, isoleucine. In 6-well plates, NPs were starved for 2 h in KRB without carbon sources and then incubated in DM ± 2 mM glutamine for 8 h. After incubation, conditioned DM was harvested, and cells were washed once with cold PBS, quenched with 500 μl of methanol (−60 °C), and scraped from the wells. Cell pellet samples were disrupted, extracted, and pretreated using the optimized MEPS protocol to ensure efficient clean-up and preconcentration [37]. The HPLC-MS/MS analysis was performed on a Waters Alliance e2695 system with autosampler coupled to a Waters Micromass Quattro Micro triple-quadrupole mass spectrometer equipped with an electrospray ion source (ESI) (Waters). MEPS-HPLC-MS/MS analysis employed multiple reaction monitoring (MRM) under both positive and negative electrospray ionization (ESI+/−) in polarity switching mode. Data were processed with Waters MassLynx 4.1 software (Waters). Samples were analyzed in triplicate, and metabolite concentrations were expressed as ng/10^6^ cells.

### RNA-sequencing and differential gene expression analysis

Bulk RNA-seq libraries were prepared by enriching mRNA from total RNA using the BGI Optimal Dual-mode mRNA Library Prep kit (LR00R96, BGI) and sequenced on an MGI DNBseq G400 PE150 platform (BGI, China). Statistical analysis was performed using R 4.4.1 and Bioconductor 3.20. All dependencies are reported in the “renv.lock” file in the paper repository on GitHub (https://github.com/Nbalb/AGC1_iPSCs), along with all analysis scripts. Reads were aligned to the human genome (GRCh38.p12) using HISAT for genome-level mapping, followed by separate alignment to reference genes using Bowtie2 to generate gene counts. Differential expression in patients (P1, P2A, and P2B) vs controls (C1, C2) was analyzed using DESeq2 [78]. Downregulated genes were defined as those with fold change (FC) < −2 (log2FC = −1) and upregulated genes as FC > 2 (log2FC = 1). Adjusted *P*-values < 0.05 were considered significant. Log2FC were shrunk using Bayesian estimator apeglm [79], and *P*-values associated with fold changes were adjusted for false discovery rate (FDR) using the Benjamini-Hochberg correction. Normalized counts were obtained via rlog transformation to remove dependence of variance on the mean and account for library size. Gene Set Enrichment Analysis (GSEA) was performed using the *fgsea* package, with gene sets obtained from the Broad Institute’s MSigDb collection via the *msigdbr* package [80].

## Supplementary information

Supplementary Figure S1 high resolution

Supplementary Figure S2 high resolution

Supplementary Figure S3 high resolution

Supplementary Figure S4 high resolution

Supplementary Figure S5 high resolution

Supplementary Figure S6 high resolution

Supplementary Figure S7 high resolution

Supplementary Figure S8 high resolution

Supplementary Figure S9 high resolution

Supplementary Figure S10 high resolution

Legends to Supplementary Figures

Supplementary Methods

Supplementary Material 1 for Fig. S1H S1I

Supplementary Material 2 for Fig. 1C

Supplementary Material 3 for Fig. 1h_1I

Supplementary Material 4 for Fig. 6J

Supplementary Material 5 for Fig. 8

Supplementary Material 6 for Fig. 8C

Supplementary Material 7 for Fig. 8D

Supplementary Material 8 for Fig. 8E

## Data Availability

Further information, including Supplementary Methods or data regarding the verification of iPSC generation, is available in the Supplementary digital content or from the corresponding authors upon reasonable request.
