# Supplementary figures and images for: Combined ketone body and glutamine supplementation restores aerobic energy production in AGC1-deficient neuronal progenitors
Source: Cell Death Dis. 2025 Dec 15;17(1):120. doi: 10.1038/s41419-025-08314-4 (PMC12848005; doi:10.1038/s41419-025-08314-4)

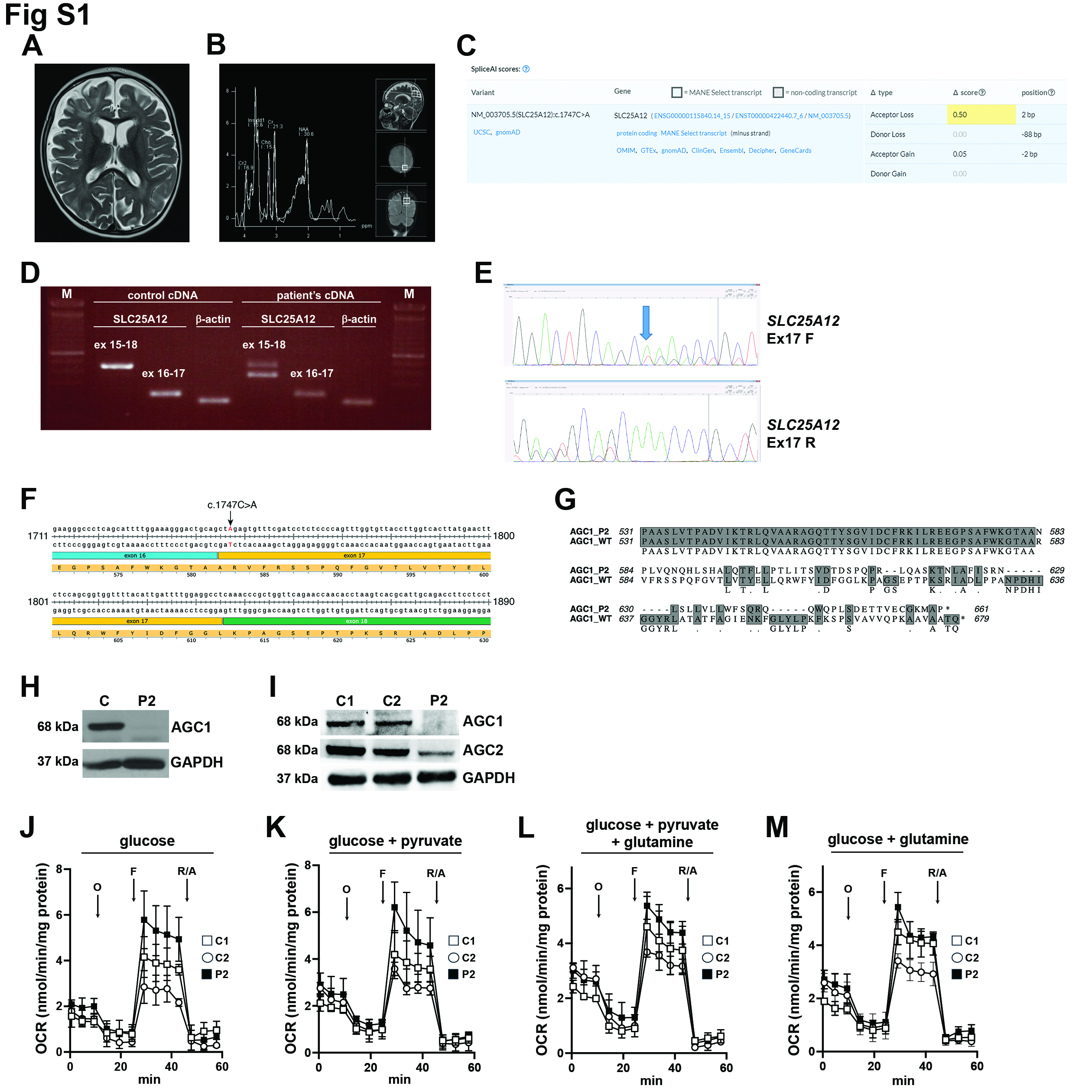

Supplement: Supplementary file 1 — Supplementary Figure S1 high resolution [file 41419_2025_8314_MOESM1_ESM.tif]

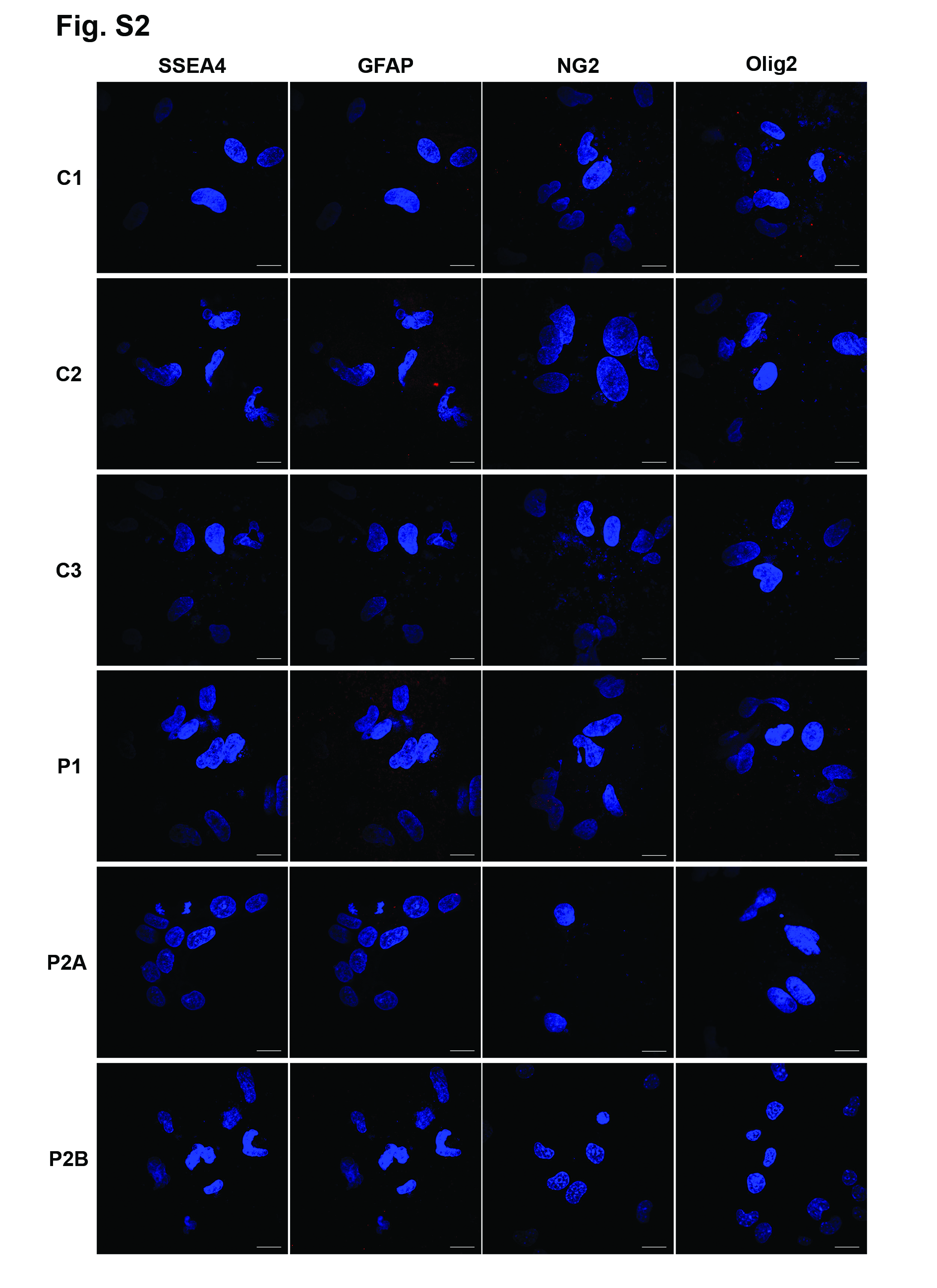

Supplement: Supplementary file 2 — Supplementary Figure S2 high resolution [file 41419_2025_8314_MOESM2_ESM.tif]

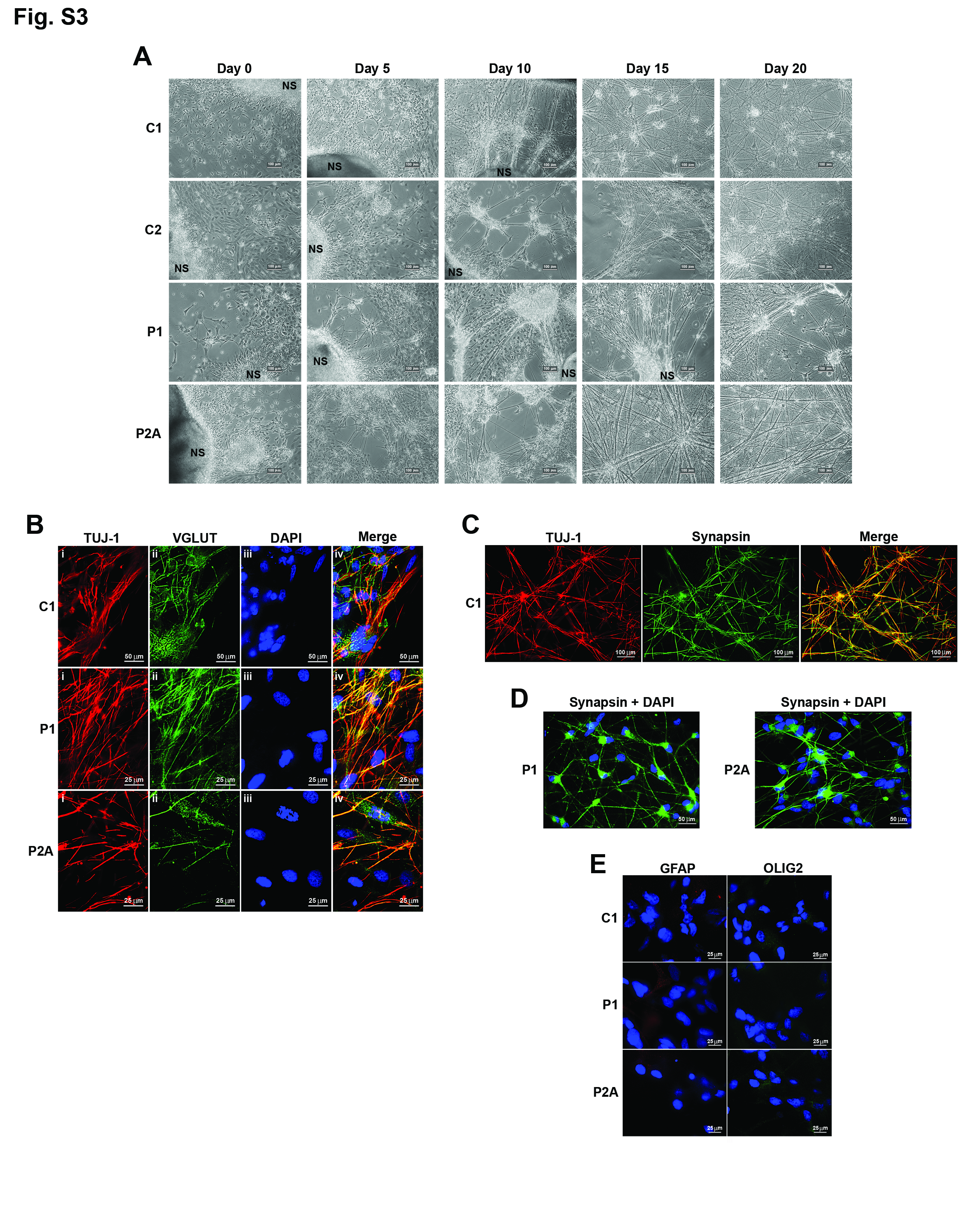

Supplement: Supplementary file 3 — Supplementary Figure S3 high resolution [file 41419_2025_8314_MOESM3_ESM.tif]

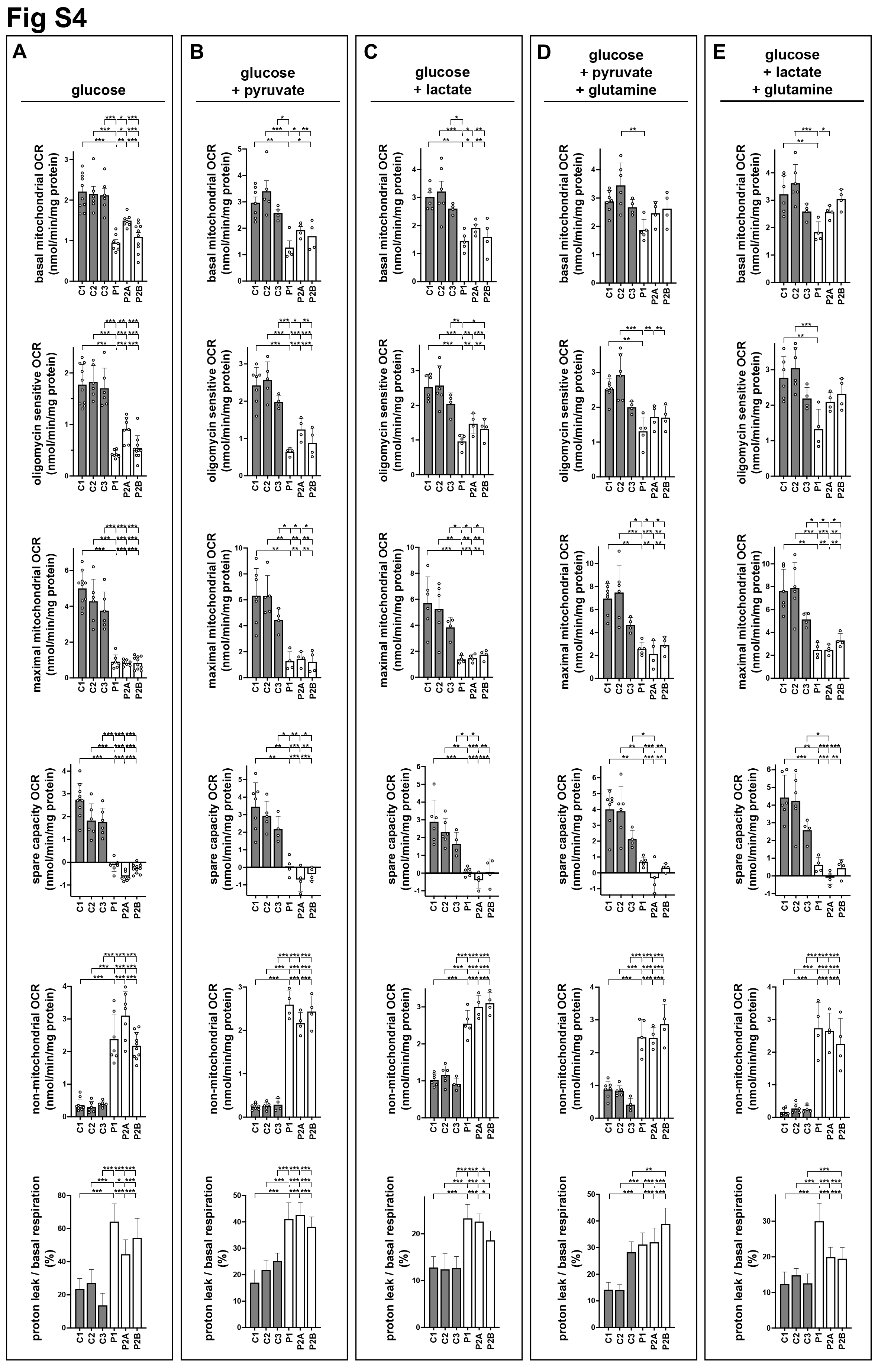

Supplement: Supplementary file 4 — Supplementary Figure S4 high resolution [file 41419_2025_8314_MOESM4_ESM.tif]

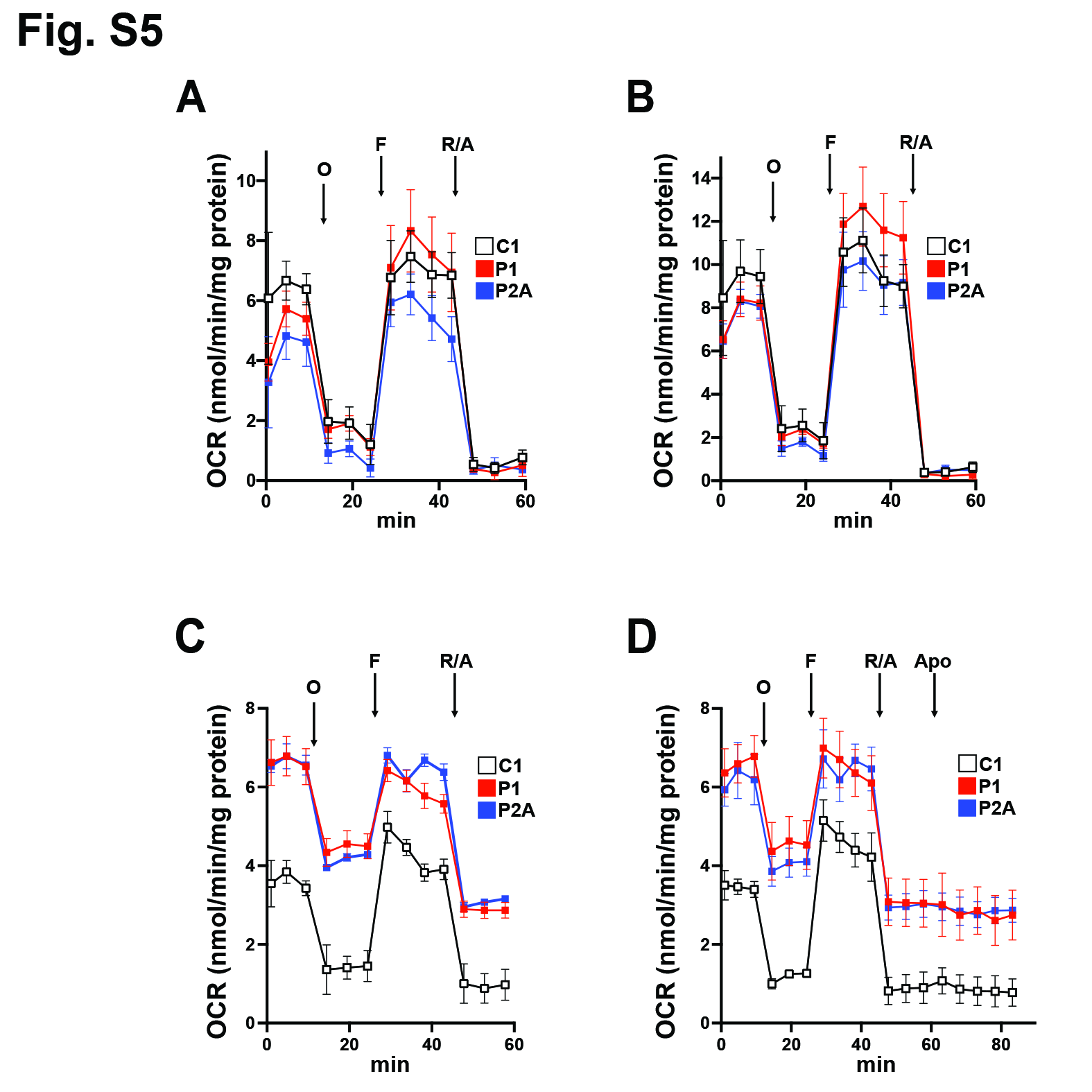

Supplement: Supplementary file 5 — Supplementary Figure S5 high resolution [file 41419_2025_8314_MOESM5_ESM.tif]

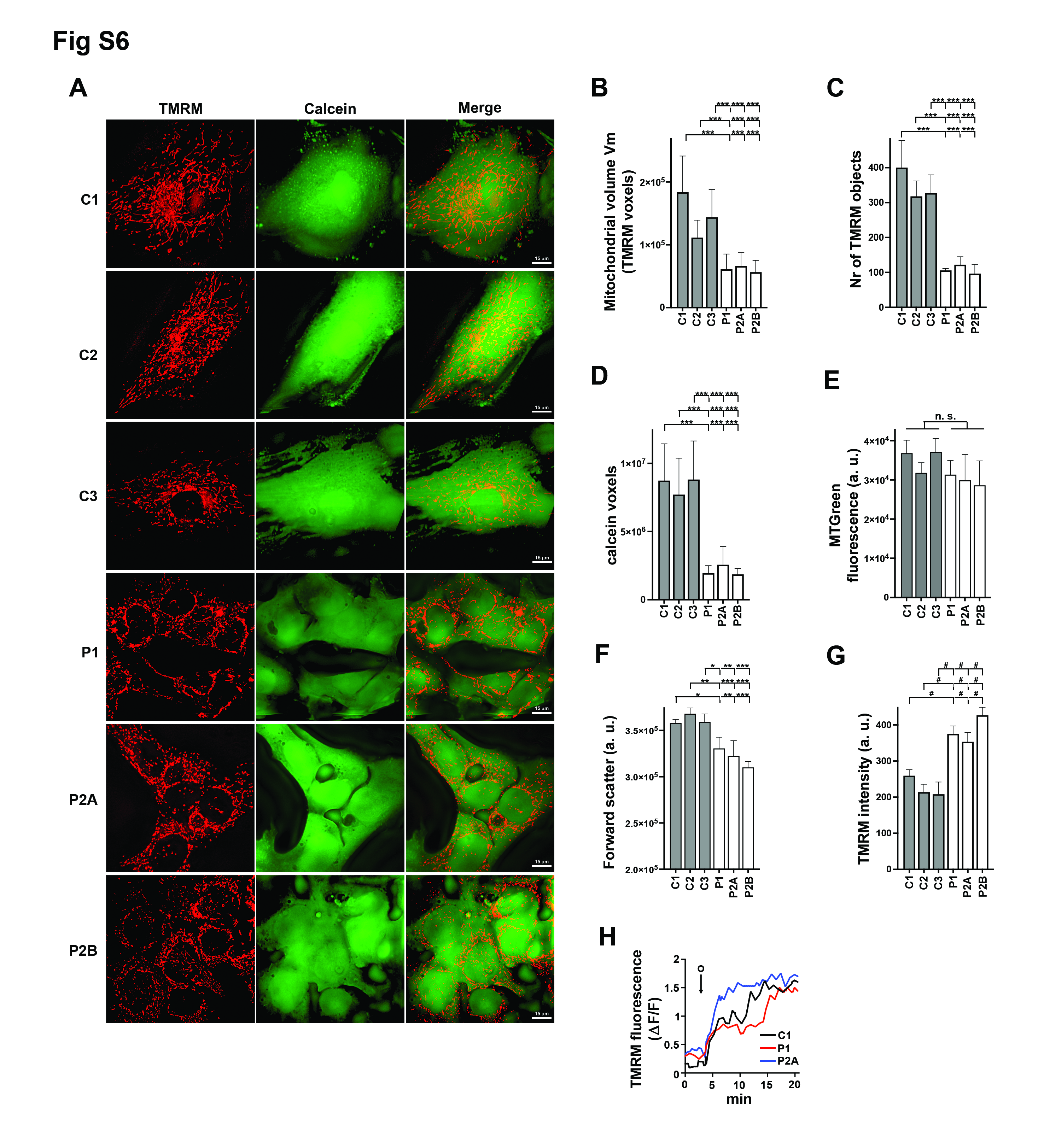

Supplement: Supplementary file 6 — Supplementary Figure S6 high resolution [file 41419_2025_8314_MOESM6_ESM.tif]

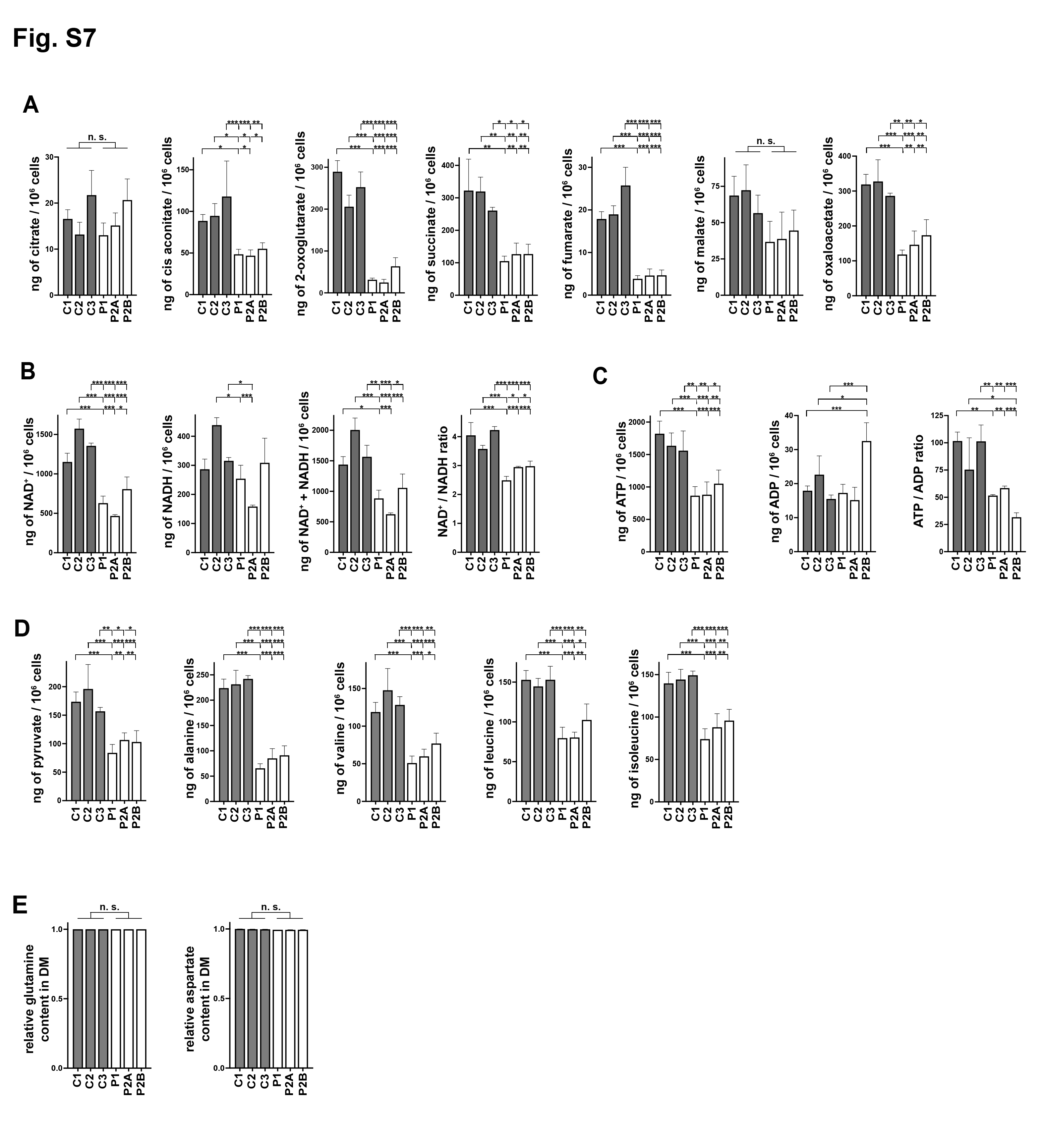

Supplement: Supplementary file 7 — Supplementary Figure S7 high resolution [file 41419_2025_8314_MOESM7_ESM.tif]

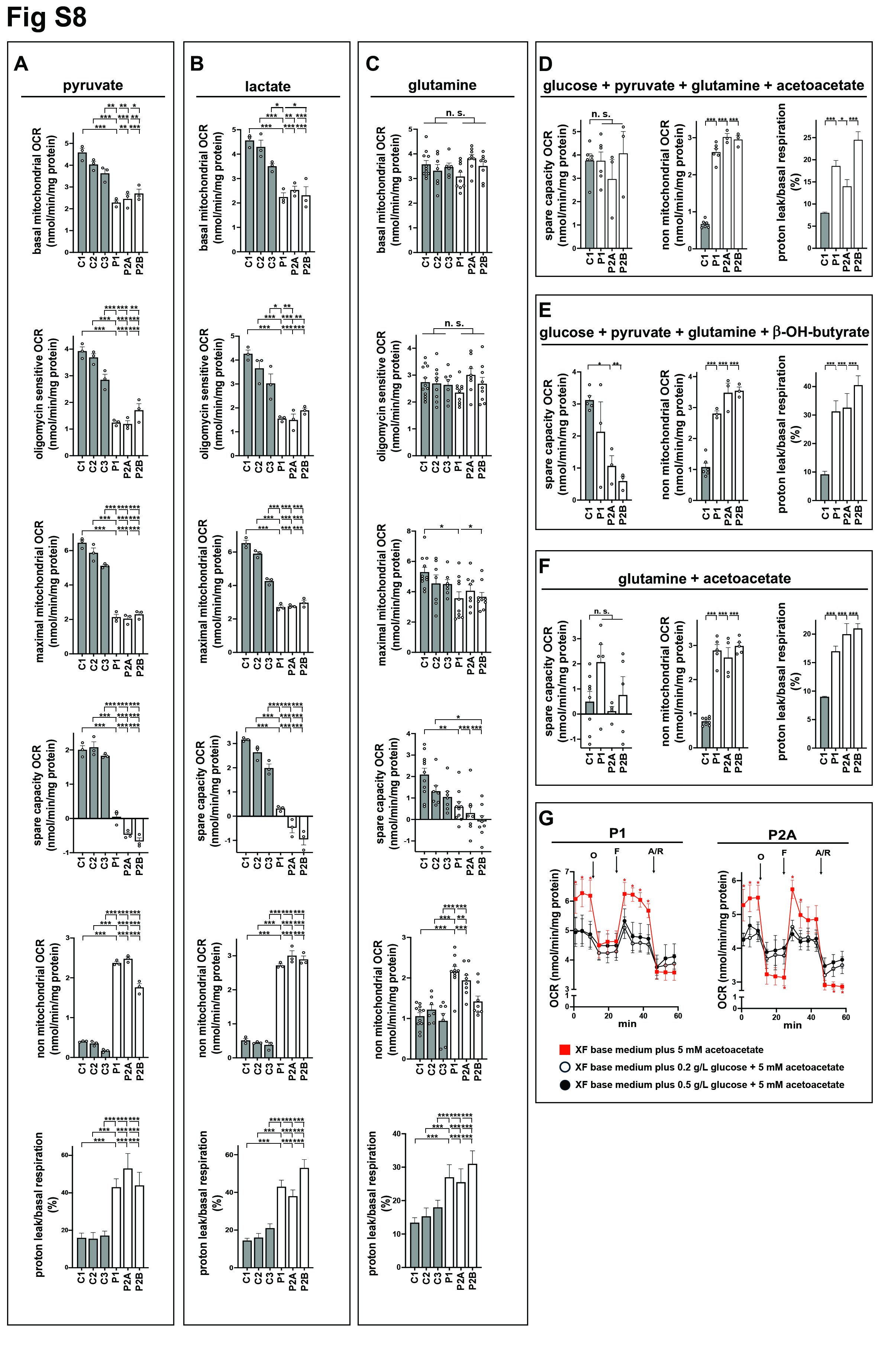

Supplement: Supplementary file 8 — Supplementary Figure S8 high resolution [file 41419_2025_8314_MOESM8_ESM.tif]

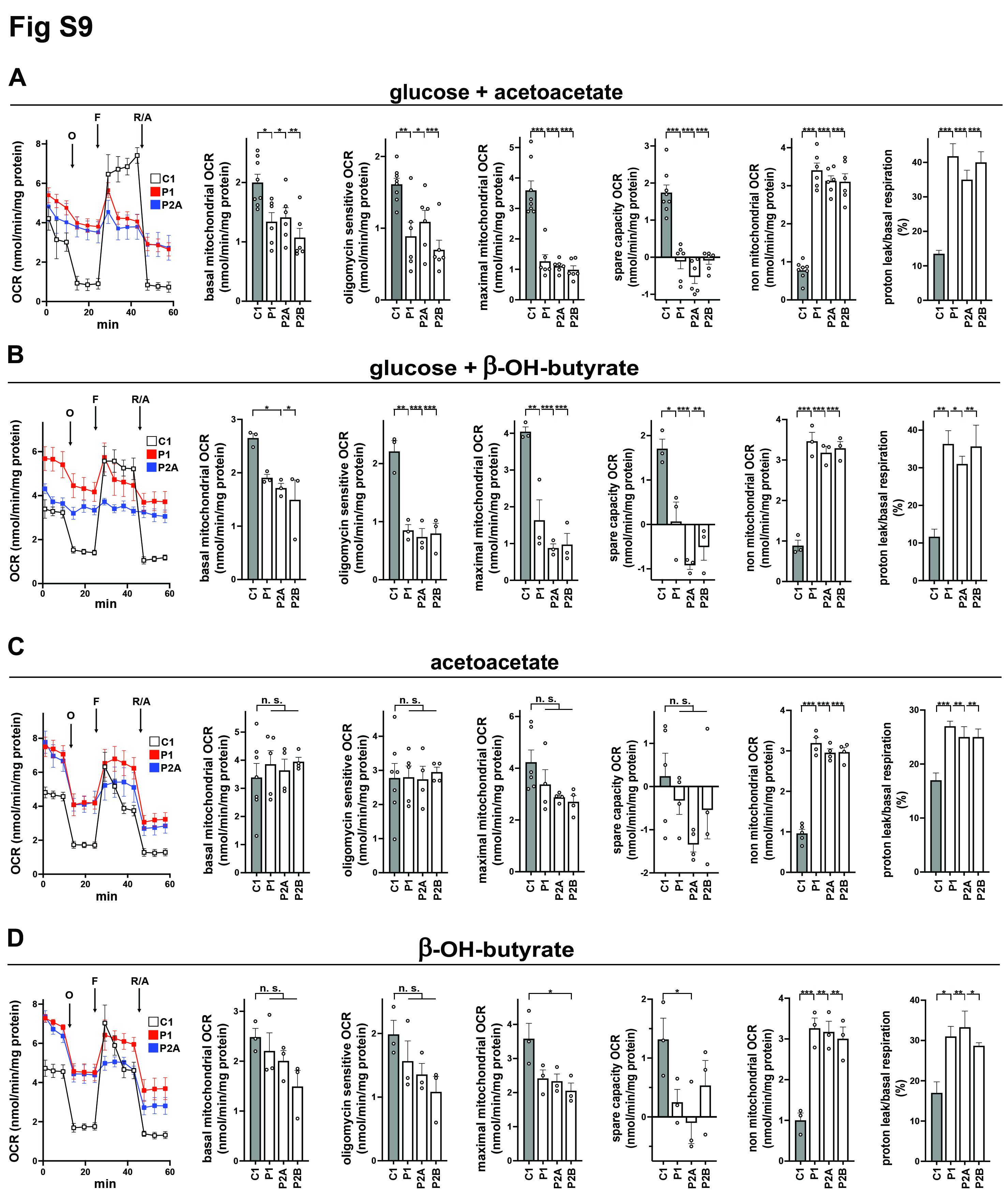

Supplement: Supplementary file 9 — Supplementary Figure S9 high resolution [file 41419_2025_8314_MOESM9_ESM.tif]

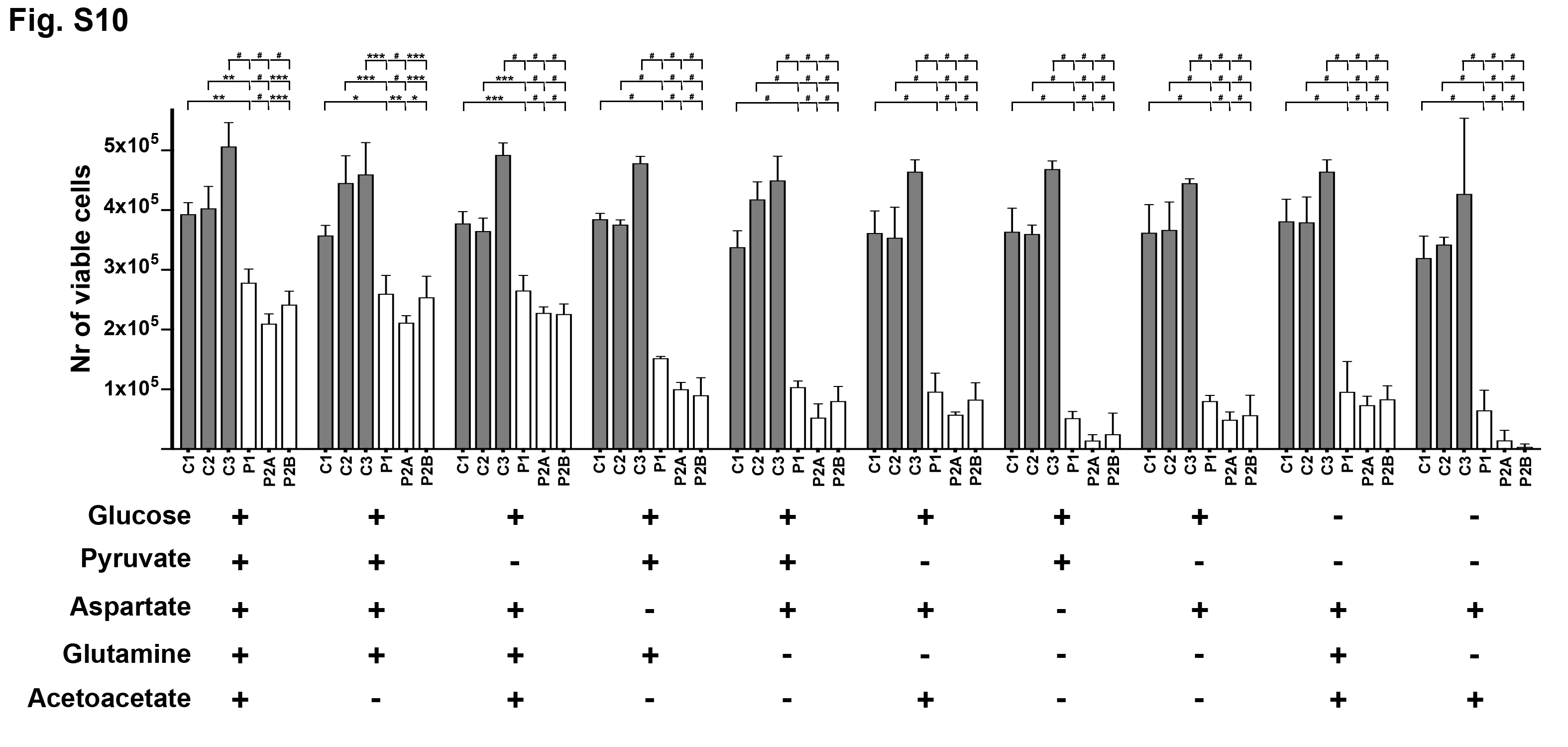

Supplement: Supplementary file 10 — Supplementary Figure S10 high resolution [file 41419_2025_8314_MOESM10_ESM.tif]

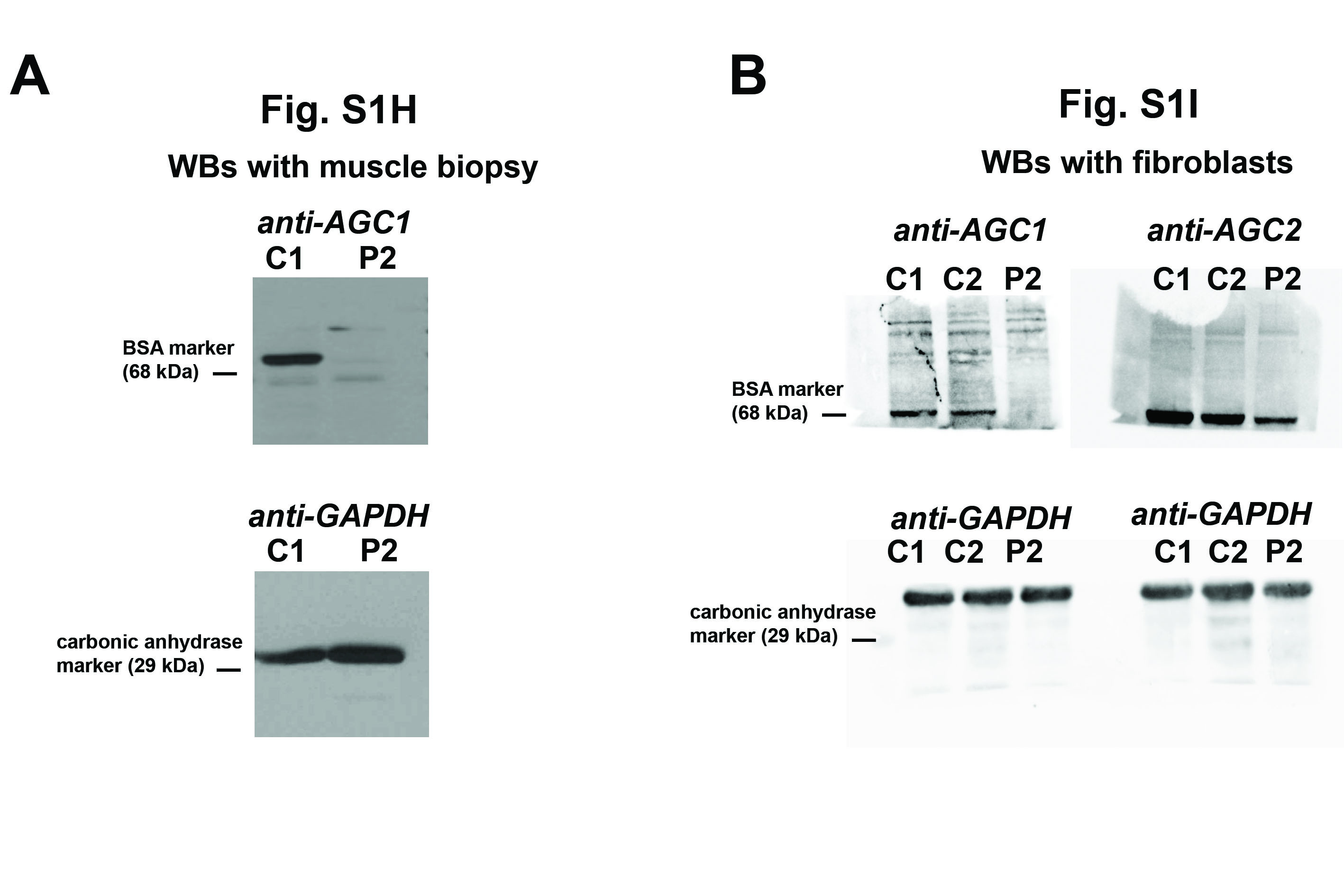

Supplement: Supplementary file 13 — Supplementary Material 1 for Fig. S1H S1I [file 41419_2025_8314_MOESM13_ESM.jpg]

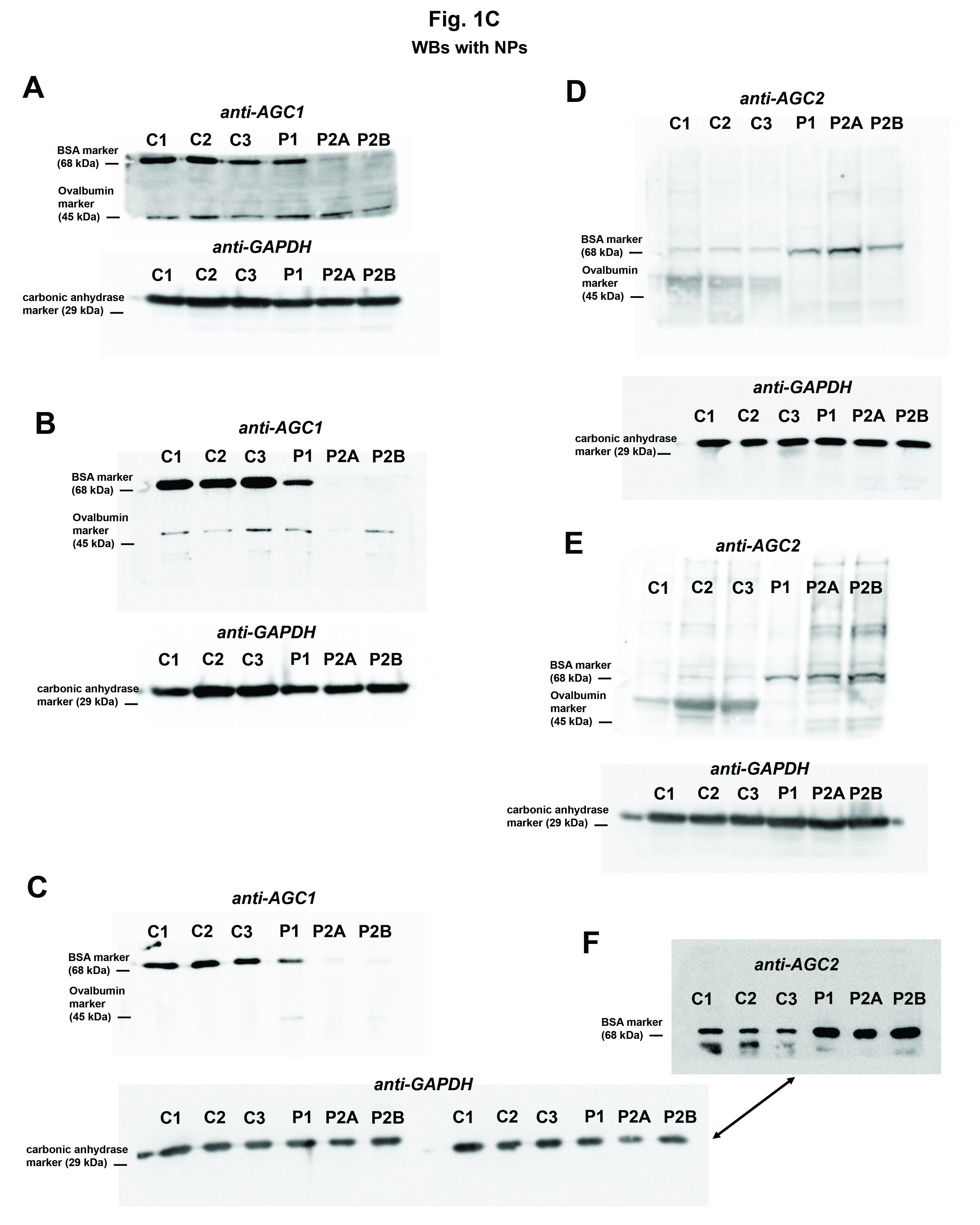

Supplement: Supplementary file 14 — Supplementary Material 2 for Fig. 1C [file 41419_2025_8314_MOESM14_ESM.jpg]

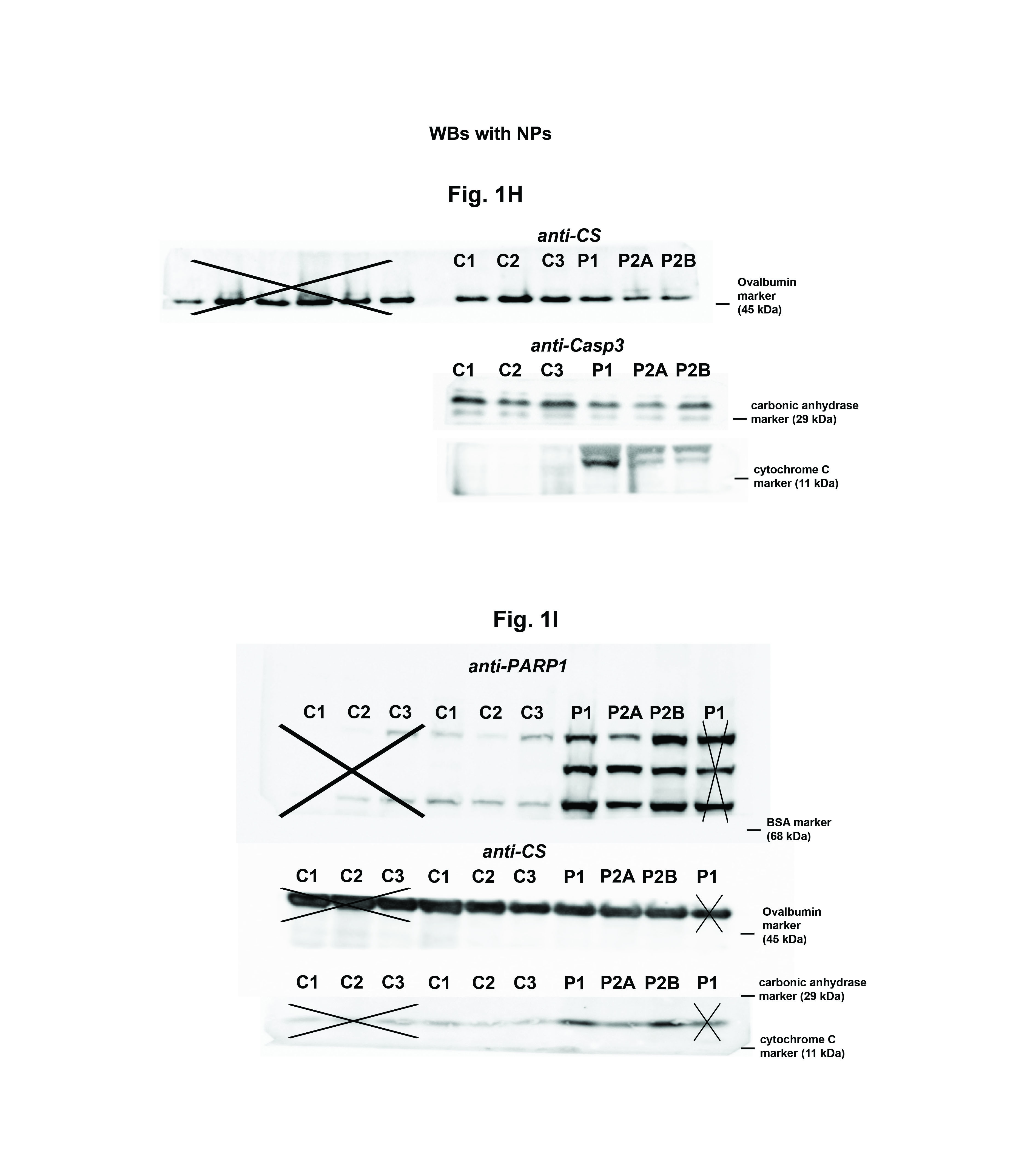

Supplement: Supplementary file 15 — Supplementary Material 3 for Fig. 1h_1I [file 41419_2025_8314_MOESM15_ESM.jpg]

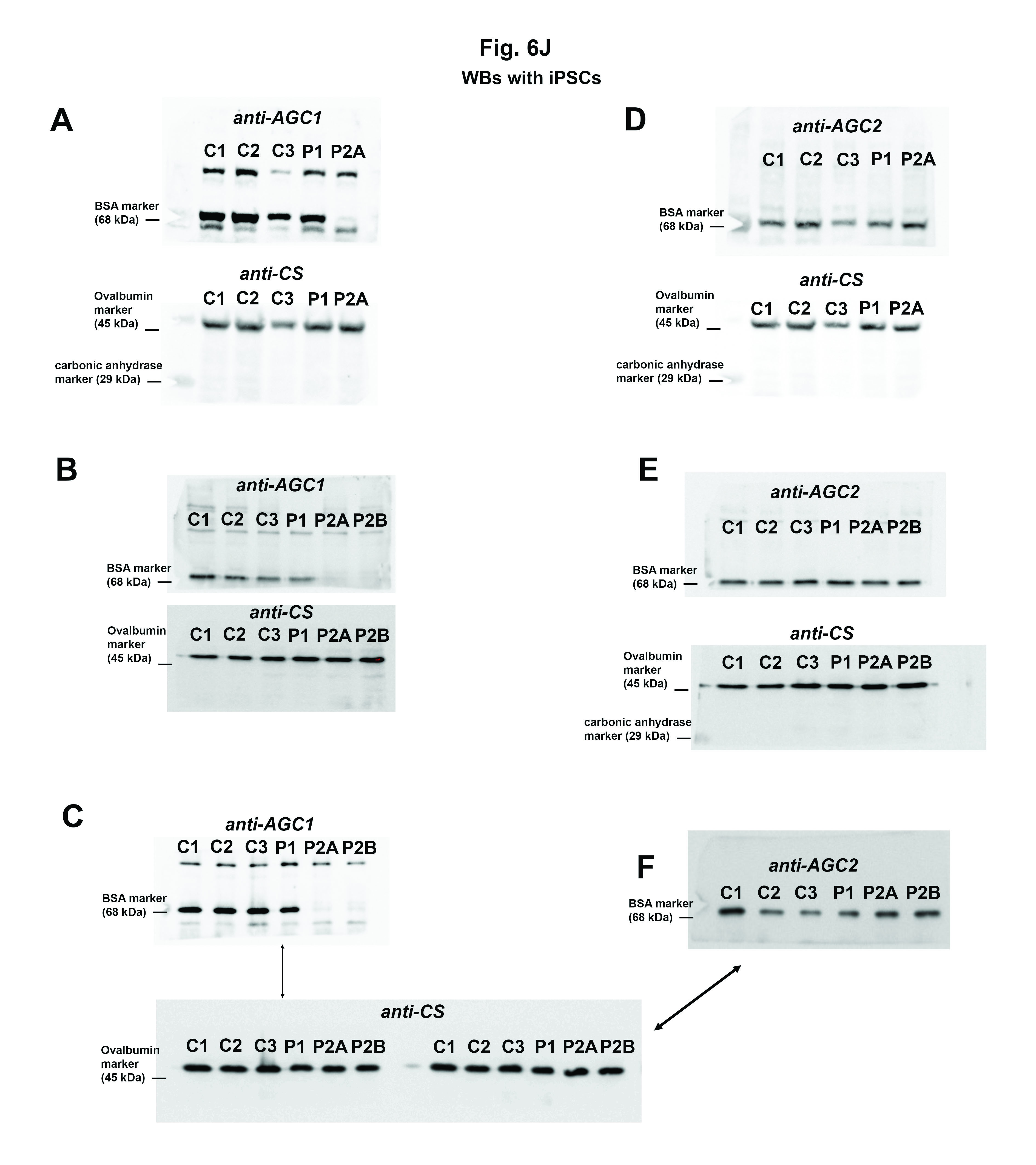

Supplement: Supplementary file 16 — Supplementary Material 4 for Fig. 6J [file 41419_2025_8314_MOESM16_ESM.jpg]

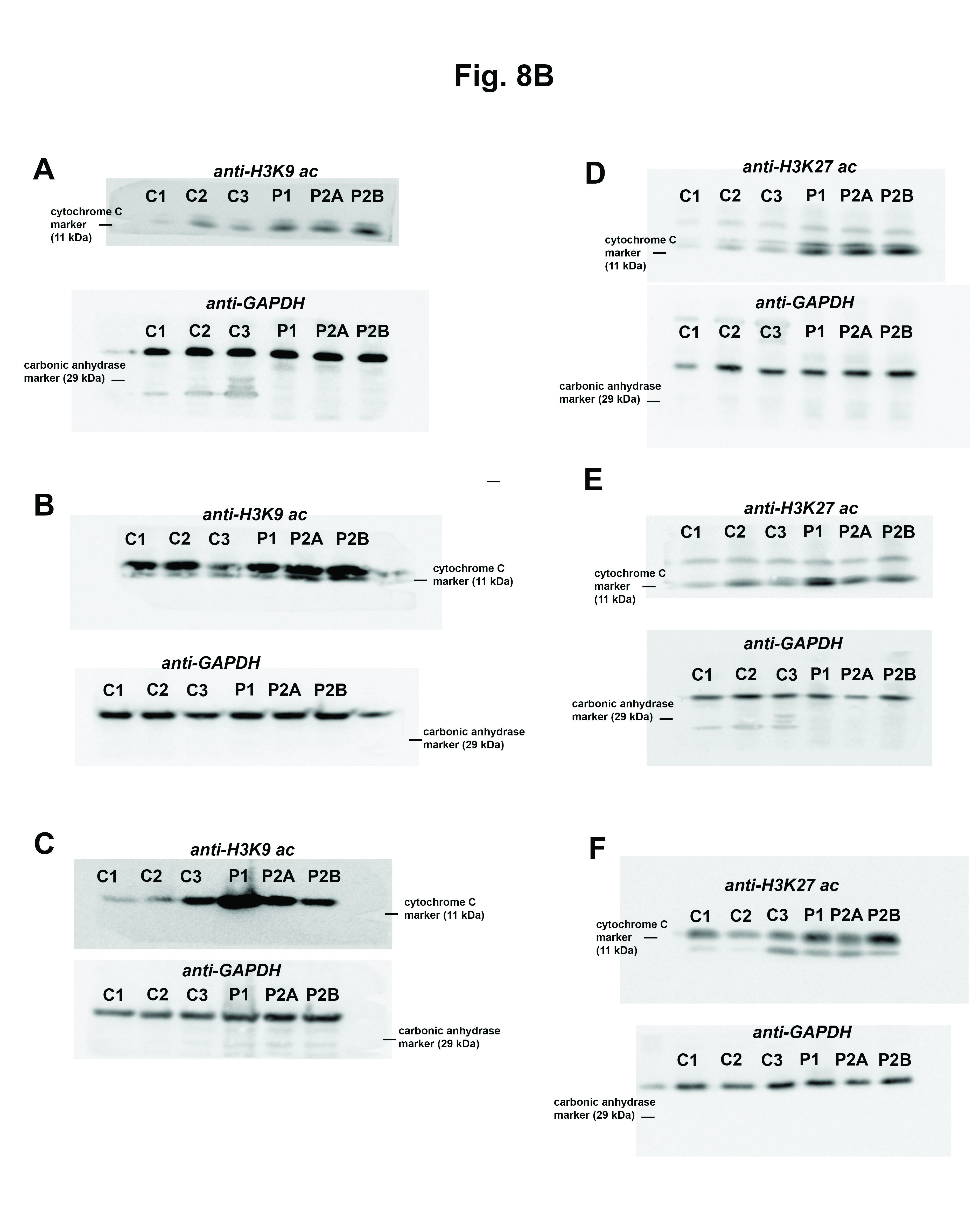

Supplement: Supplementary file 17 — Supplementary Material 5 for Fig. 8 [file 41419_2025_8314_MOESM17_ESM.jpg]

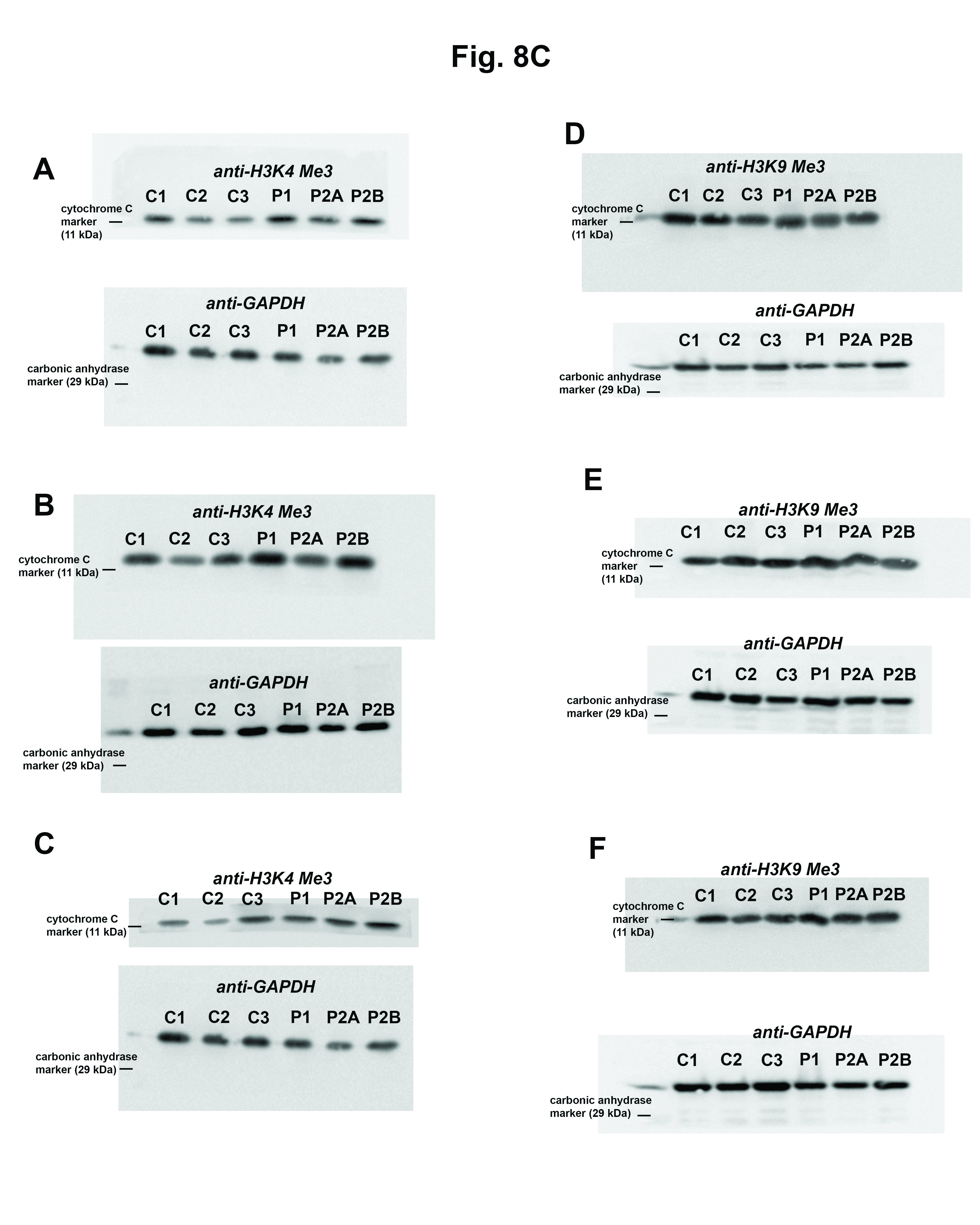

Supplement: Supplementary file 18 — Supplementary Material 6 for Fig. 8C [file 41419_2025_8314_MOESM18_ESM.jpg]

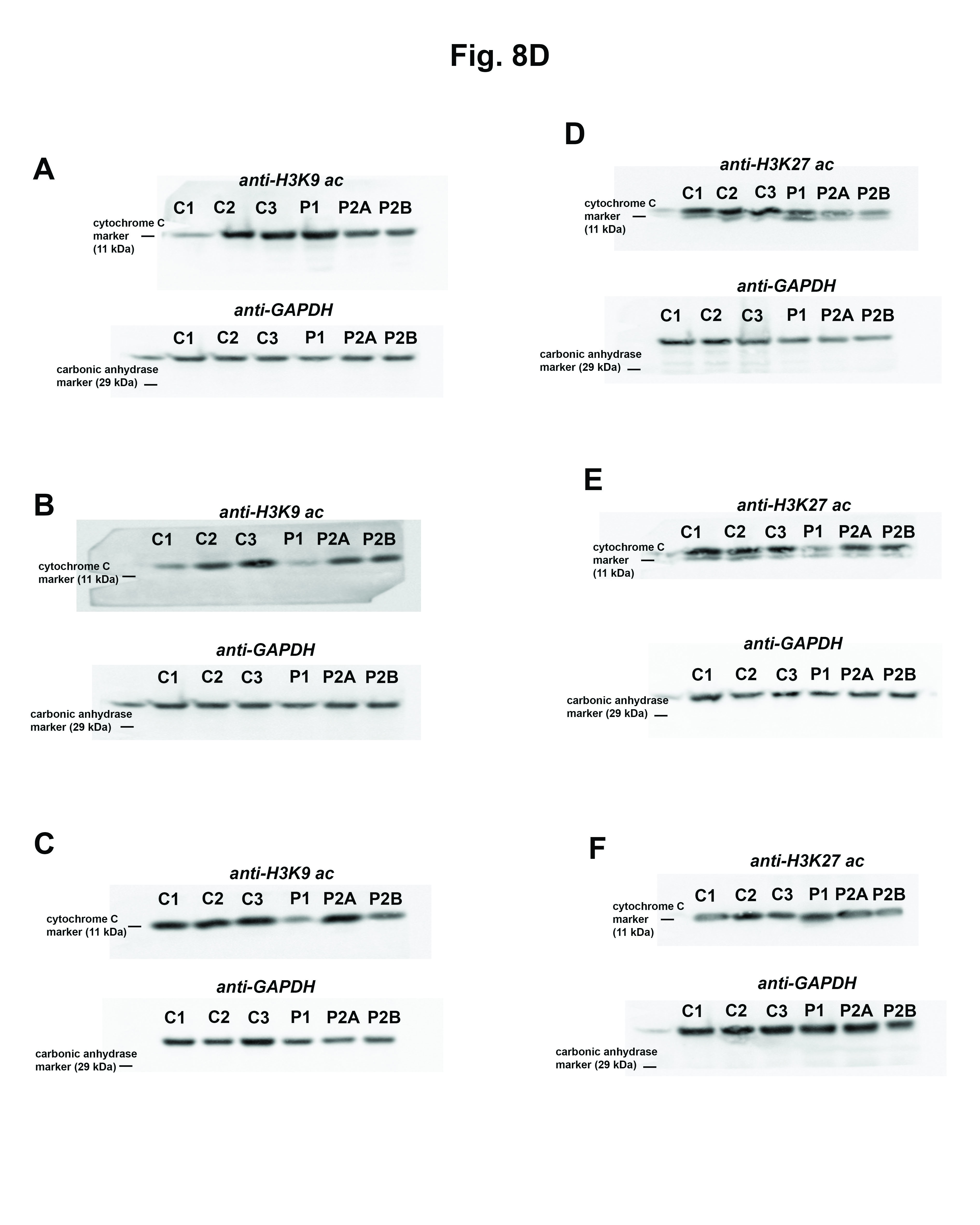

Supplement: Supplementary file 19 — Supplementary Material 7 for Fig. 8D [file 41419_2025_8314_MOESM19_ESM.jpg]

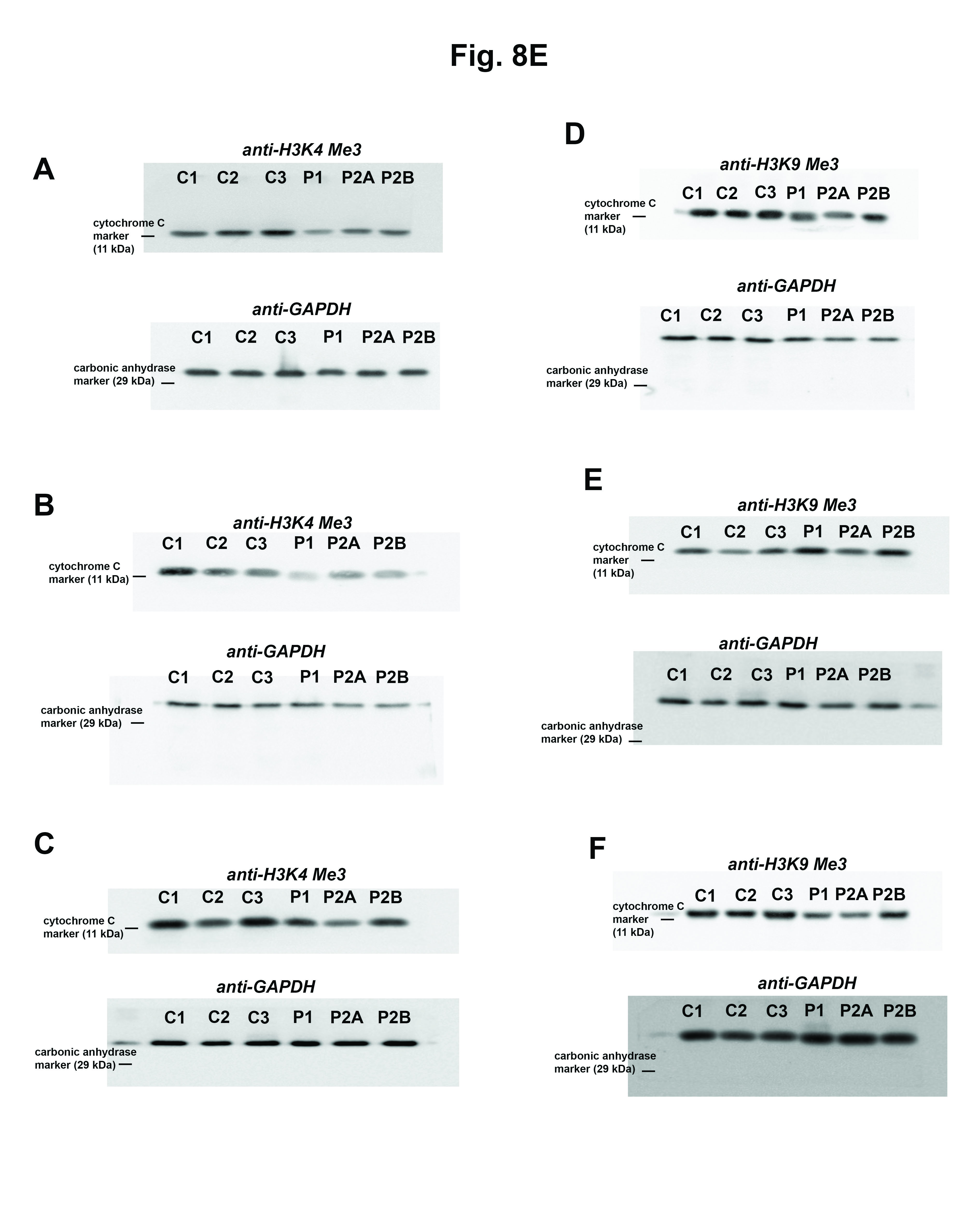

Supplement: Supplementary file 20 — Supplementary Material 8 for Fig. 8E [file 41419_2025_8314_MOESM20_ESM.jpg]
