# Supplementary material for: Combined ketone body and glutamine supplementation restores aerobic energy production in AGC1-deficient neuronal progenitors
Source: Cell Death Dis. 2025 Dec 15;17(1):120. doi: 10.1038/s41419-025-08314-4 (PMC12848005; doi:10.1038/s41419-025-08314-4)
Supplement: Supplementary file 11 — Legends to Supplementary Figures [file 41419_2025_8314_MOESM11_ESM.docx]

**LEGENDS TO SUPPLEMENTARY FIGURES.**

**Fig. S1. The c.1747C>A mutation in patient 2 (P2) causes skipping of exon 17 in *SLC25A12*.** (A) Axial T2-weighted MRI scan of P2’s brain showing moderate widening of internal and external CSF spaces. (B) MR-spectroscopy showing myoinositol and reduced NAA-peaks. (C) SpliceAI analysis revealing that the synonymous variant c.1747C>A; p.(=) is predicted to generate a new cryptical splice site in *SLC25A12*. (D) PCR analysis of first-strand cDNA synthesized from RNA extracted from the patient’s blood, surrounding the mutation reveals a heterozygous loss of a small fragment between exons 15 and 18 in P2. (E) Sanger sequencing of P2’s AGC1 cDNA confirms heterozygous exon 17 skipping. (F) The cDNA sequence of P2’s AGC1, encompassing exons 16, 17, and 18, highlights the c.1747>A mutation. (G) Amino acid sequence alignment of WT AGC1 with P2’s AGC1, resulting from exon 17 skipping, reveals a frameshift starting at position 583, leading to an aberrant C-terminal domain beginning from the sixth transmembrane α-helix (7). WB analysis confirmed the null expression of AGC1 in (H) 30 μg of muscle biopsies and (I) 100 μg fibroblasts of P2 lysed and used to react with antibodies raised against the indicated proteins (4). (J-M) Oxygen consumption rates (OCR) were measured with Seahorse XFe96 extracellular flux analyzer in fibroblasts of P2 (black squares) and fibroblasts of two unrelated healthy controls (white symbols). Fibroblasts were first incubated for 2 h in XF base medium supplemented with (J) 1 g/L glucose, (K) 1 g/L glucose + 1 mM pyruvate, (L) 1 g/L glucose + 1 mM pyruvate + 2 mM glutamine, or (M) 1 g/L glucose + 2 mM glutamine. Then cells were exposed to sequential additions of 2 μM oligomycin O, 0.5 μM FCCP F, and 1 μM rotenone + 1 μM antimycin A R/A. Shown graphs are representative of three independent experiments each including 4–5 replicates per cell type and revealing non-significant OCR deficit of patient fibroblasts in terms of mitochondrial basal respiration, ATP turn over and maximal respiration [33].

**Fig. S2. NPs from control and AGC1 deficiency patients show no evidence of non-neuronal differentiation.** NPs from controls (C1, C2, C3) and patients (P1, P2A, P2B) were immunostained for markers of stem cells (stage-specific embryonic antigen 4, SSEA4), astroglia (GFAP), OPCs (NG2) or oligodendroglia (Olig2). Nuclei were labelled with 1 μg/mL DAPI. Confocal images acquired using a 100X objective revealed no positive cells for the analyzed markers, excluding a non-neuronal differentiation fate for the investigated NPs. For each experiment shown, three different fields were acquired which were identical for SSEA4 and GFAP co-immunostaining performed with green- and red-labelled secondary antibodies, respectively; bar scale: 10μm.

**Fig. S3. AGC1-deficient neurospheres during the early stages of differentiation into mature neurons.** (A) Bright-field images of neurospheres (NS) generated from control (C1 and C2) and patient-derived hiPSCs (P1 and P2A) during a 20-day incubation in Neurobasal differentiation medium supplemented with 2% MACS NeuroBrew-21 and 10 ng/ml human BDNF to stimulate neurite outgrowth. Images were acquired at the indicated time points using a 20X objective on a Nikon Eclipse TS100 microscope. (B) Fluorescence images of C1, P1 and P2A NS-derived neurons immunostained for TUJ1 (red panels) and VGLUT (green panels). Nuclei were counterstained with 1 μg/mL DAPI. (C) Fluorescence images of C1 NS-derived neurons immunostained for TUJ1 (red panels) and Synapsin (green panels). (D) P1 and P2 NS-derived neurons immunostained for Synapsin and labelled with 1 μg/mL DAPI. Images were acquired using a 40X objective on an epifluorescence Zeiss Axiovert 200 microscope equipped with a CoolSnap HQ CCD camera and deconvolved using Metamorph software. (E) C1, P1 and P2A NS-derived neurons incubated with antibodies against astroglial (GFAP) and oligodendroglial (Olig2) markers and counterstained with 1 μg/mL DAPI showed no significant immunostaining. Scale bars are indicated for each panel.

**Fig. S4. Bioenergetic parameters of NPs with AGC1 deficiency.** Mitochondrial basal respiration, oligomycin sensitive respiration (i.e, ATP turn over), maximal respiration, spare capacity, non-mitochondrial respiration, and proton leak calculated in Mitostress experiments performed with control (C1, C2, C3; grey bars) and AGC1 deficiency patient NPs (P1, P2A, P2B; white bars) incubated for 2 hours in XF base medium supplemented with (A) 1 g/L glucose, (B) 1 g/L glucose + 1mM pyruvate, (C) 1 g/L glucose + 5 mM lactate, (D) 1 g/L glucose + 1mM pyruvate + 2 mM glutamine, or (E) 1 g/L glucose + 5 mM lactate + 2 mM glutamine. Cells were then sequentially exposed to 2 μM oligomycin, 0.5 μM FCCP, and 1 μM rotenone + 1 μM antimycin A, as shown in figures 2 G-K, and bioenergetic parameters were measured as previously described [33]. Data are means ± SD of at least four independent experiments, each including 5–6 replicates per cell type. *p<0.05, **p<0.01, ***p<0.001 by one-way ANOVA with Tukey’s comparison test.

**Fig. S5**. (A-B) Mitochondrial respiration is not altered in hiPSCs from patients with AGC1 deficiency. Representative Mito Stress experiments for control C1 (white squares), patient P1 (red squares) and P2A (blue squares) hiPSCs incubated for 2 hours in XF base medium supplemented with (A) 1 g/L glucose + 1 mM pyruvate, or (B) 1 g/L glucose + 1 mM pyruvate + 2 mM glutamine. Cells were then exposed to sequential additions (arrows) of 2 μM oligomycin O, 0.5 μM FCCP F, and 1 μM rotenone + 1 μM antimycin A R/A. Each data point represents the mean ± SD of 4replicates per cell type. (C-D) Apocynin is ineffective in reducing non-mitochondrial respiration in AGC1-deficient NPs. Representative Mito Stress experiments for control C1 (white squares), patient P1 (red squares) and P2A (blue squares) NPs incubated for 2 hours in XF base medium supplemented with 1 g/L glucose + 1 mM pyruvate + 2 mM glutamine. Cells were then exposed to sequential additions (arrows) of 2 μM oligomycin O, 0.5 μM FCCP F, and 1 μM rotenone + 1 μM antimycin A R/A. 0.5 mM of the NADPH oxidase inhibitor apocynin was added either (C) during the 2-hour incubation in XF medium or (D) after the addition of rotenone + antimycin at the end of the measurement (Apo arrow), without affecting the non-mitochondrial OCR measured. Each data point represents the mean ± SD of 4–5 replicates per cell type. The shown graphs are representative of at least two independent experiments that produced similar results.

**Fig. S6. Impact of AGC1 deficiency on mitochondrial network architecture and biogenesis.** (A) Representative maximum intensity projection images of control (C1, C2, C3) and patient NPs (P1, P2A, P2B) stained with 1 µM Calcein-AM (green) and 2 nM TMRM (red). These images were used to obtain the morphometric analysis presented in Fig. 3. Scale bars represent 10 µm. (B - D) The total mitochondrial volume (B, TMRM voxels), the number of mitochondria (C, TMRM objects), and the cellular volume (D, calcein voxels) of control (C1, C2, C3; grey bars) and patient NPs (P1, P2A, P2B; white bars) were calculated using surface rendering of TMRM or Calcein-AM staining. (E) Mitochondrial mass and (F) cellular size of control (C1, C2, C3; grey bars) and patient NPs (P1, P2A, P2B; white bars) were quantified by flow cytometry, by measuring the fluorescence intensity of the cells loaded with 100nM MitoTrackerGreen and their forward scatter, respectively. (G) ΔΨm was measured in deconvolved images of control (C1, C2, C3; grey bars) and patient NPs (P1, P2A, P2B; white bars) incubated for 2 h in DM supplemented with 1 g/L glucose, 1 mM pyruvate, 2 mM glutamine and loaded with 2 nM TMRM. (H) Representative traces of TMRM fluorescence intensities after addition of 1.5 µM oligomycin (arrow) measured in control (C1, black line) and patient NPs (P1 and P2A, red and blue line respectively) incubated as indicated in (G). Data are the means ± SD from at least three independent experiments. *p<0.05, **p<0.01, ***p<0.001, ^#^p<0.0001 by one-way ANOVA with Tukey’s test.

**Fig. S7.** (A-D) Metabolite content in NPs with AGC1 deficiency grown in the absence of glutamine. Control (C1, C2, C3; grey bars) and patient NPs (P1, P2A, P2B; white bars) were harvested after 8 hours of incubation with DM without glutamine supplementation. The total cellular pools of (A) TCA cycle metabolites - citrate, cis-aconitate, 2-oxoglutarate, succinate, fumarate, L-malate and oxaloacetate; (B) NAD^+^, NADH, and relative NAD^+^/NADH ratio, (C) ATP, ADP and relative ATP/ADP ratio and (D) pyruvate, alanine, valine, leucine, and isoleucine were determined by HPLC-MS/MS analysis as detailed in Methods section. (E) Relative content of glutamine and aspartate in conditioned DM of control (C1, C2, C3; grey bars) and patient NPs (P1, P2A, P2B; white bars) harvested after 8 hours of incubation in complete DM and quantified by LC-MS/MS analysis, as detailed in Methods. Data are the means ± SD of four independent preparations; *p<0.05, **p<0.01, ***p<0.001, from one-way ANOVA with Tukey’s test.

**Fig. S8.** (A-C) Mitochondrial basal respiration, oligomycin sensitive respiration (i.e, ATP turn over), maximal FCCP sensitive respiration, spare capacity, non-mitochondrial respiration, and proton leak calculated in Mito Stress experiments represented in figures 6K-M performed with control (C1, C2, C3; grey bars) and AGC1 deficiency patient NPs (P1, P2A, P2B; white bars) incubated for 2 hour in XF base medium supplemented with (A) 1mM pyruvate, (B) 5 mM lactate, or (C) 2mM glutamine. (D-F) Spare capacity, non-mitochondrial respiration, and proton leak calculated in Mito Stress experiments represented in figures 7 A-C performed with control (C1; grey bars) and AGC1 deficient NPs (P1, P2A, P2B; white bars) incubated for 2 hour in XF base medium supplemented with (D) 1 g/L glucose + 1 mM pyruvate + 2 mM glutamine + 5 mM acetoacetate, (E) 1 g/L glucose + 1 mM pyruvate + 2 mM glutamine + 5 mM β-OH-butyrate, or (F) 2 mM glutamine + 5 mM acetoacetate. Oxygen consumption rates (OCR) were measured before and after sequential exposure to 2 μM oligomycin, 0.5 μM FCCP, and 1 μM rotenone + 1 μM antimycin A. Bioenergetic parameters were measured as previously described (33). Proton leak was expressed as a percentage of mitochondrial basal respiration. Data are the means ± SD of at least three independent experiments, each including 4-5 replicates per cell type. *p<0.05, **p<0.01, ***p<0.001 by one-way ANOVA with Tukey’s test. (G) Glucose inhibits the recovery of mitochondrial respiration mediated by KB in NPs with AGC1 deficiency. Mito Stress experiments performed with AGC1-deficient NPs P1 and P2A incubated for 2 hours in XF base medium supplemented with 5 mM acetoacetate (red squares), 0.2 g/L glucose + 5 mM acetoacetate (white circles), or 0.5 g/L glucose + 5 mM acetoacetate (black circles). Cells were then exposed to sequential additions (arrows) of 2 μM oligomycin O, 0.5 μM FCCP F, and 1 μM rotenone + 1 μM antimycin A R/A. Data are the means ± SD at the corresponding time-point from 4-5 replicates per cell type. *p<0.05 compared to NPs incubated with 5 mM acetoacetate.

**Fig. S9. Glucose inhibits the recovery of mitochondrial respiration mediated by ketone bodies in NPs with AGC1 deficiency.** (A-D) Representative OCR traces and calculated mitochondrial basal respiration, oligomycin sensitive respiration (i.e, ATP turn over), maximal FCCP sensitive respiration, spare capacity, non-mitochondrial respiration, and proton leak from Mito Stress experiments performed with control (C1, grey bars) and patient AGC1-deficient NPs (P1, P2A, P2B; white bars) incubated for 2 hours in XF base medium supplemented with (A) 1 g/L glucose + 5 mM acetoacetate, (B) 1 g/L glucose + 5 mM β-OH-butyrate, (C) 5 mM acetoacetate, or (D) 5 mM β-OH-butyrate. Cells were then exposed to sequential additions (arrows) of 2 μM oligomycin O, 0.5 μM FCCP F, and 1 μM rotenone + 1 μM antimycin A R/A, and bioenergetic parameters measured as previously described [33]. Proton leak was expressed as a percentage of mitochondrial basal respiration. Data are the means ± SD of at least three independent experiments, each including 4-5 replicates per cell type. *p<0.05, **p<0.01, ***p<0.001 by one-way ANOVA with Tukey’s test.

**Fig. S10. Comparison of cell proliferation between control and AGC1-deficient NPs in XF base media.** Trypan Blue exclusion assays were performed on control (C1, C2, C3; grey bars) and AGC1-deficient (P1, P2A, P2B; white bars) NPs, which were seeded at a density of 20,000 cells/cm² and grown for 24 hours in XF base medium supplemented with 15 mM HEPES, 10 ng/ml bFGF, 10 ng/ml EGF, 1% N2 supplement, 2% Neuro-Brew-21 w/o vitamin A, 50 ng/ml BSA, 0.5 mM Vitamin B12, 14 nM biotin, 15 μM hypoxanthine, 0.5 μM lipoic acid, 1.5 μM thymidine, 49 μM alanine, 50 μM asparagine, 100 μM cysteine, 50 μM glutamate, 150 μM proline. The medium also contained 1 g/L glucose, 1 mM pyruvate, 2 mM glutamine, 5 mM acetoacetate and 50 μM aspartate in the indicated combinations. Data are presented as the mean ± SD of at least three independent assays. One-way ANOVA with Tukey’s test was used to compare control and patient NPs; *p<0.05, **p<0.01, ***p<0.001, ^#^p<0.0001.
