# Supplementary material for: Combined ketone body and glutamine supplementation restores aerobic energy production in AGC1-deficient neuronal progenitors
Source: Cell Death Dis. 2025 Dec 15;17(1):120. doi: 10.1038/s41419-025-08314-4 (PMC12848005; doi:10.1038/s41419-025-08314-4)
Supplement: Supplementary file 12 — Supplementary Methods [file 41419_2025_8314_MOESM12_ESM.docx]

**SUPPLEMENTARY METHODS.**

**Generation of hiPSCs-derived neurospheres.**

hiPSCs were differentiated into neurospheres following the protocol of D’Aiuto et al. [32]. Briefly, hiPSC colonies dissected using the StemPro™ EZPassage™ Tool (ThermoFisher) were seeded at low density on Matrigel-coated plates and cultured in StemMACS iPS-Brew XF medium for one week. Then, medium was replaced with Neural Precursor Selection (NPS) medium, composed of DMEM/F-12 (cat. 21331020, ThermoFisher) supplemented with 0.5% N2 supplement, 2 mM L-glutamine, 1% MEM non-essential amino acids (cat. 11140035, ThermoFisher), 50 U/ml penicillin G, 50 mg/ml streptomycin (Merck), and 0.1 mM 2-mercaptoethanol (Merck). After one week, the NPS medium was replaced with the Neural Precursor Expansion (NPE) medium, consisting of DMEM/F-12 supplemented with 1% N2 supplement, 20 ng/ml Human FGF-2 IS, 2 mM L-glutamine, 1% MEM non-essential amino acids, 50 U/ml penicillin G, 50 mg/ml streptomycin, and 0.1 mM 2-mercaptoethanol. The differentiating hiPSCs were cultured for at least 7 days, leading to the formation of cellular aggregates containing neuronal rosettes that were manually dissected and transferred to ultra-low attachment plates (cat. CLS3471, Corning) to promote the formation of neurosphere-like (NL) structures. Once rounded, NL were plated on 6-well Matrigel-coated plates and over the following days productive neurospheres were transferred back to ultra-low attachment 6-well plates. The procedure was repeated for at least 3 rounds and the resulting NL were exposed to the final differentiation medium composed of Neurobasal medium supplemented with 2% MACS NeuroBrew-21 (Miltenyi, cat. 130093566), 10 ng/ml human BDNF (cat. AF-450-02, ThermoFisher), 50 U/ml penicillin G, and 50 mg/ml streptomycin for 20 days to achieve near-complete differentiation to neurosphere-derived neurons.

**Western blot analysis.**

Cells were lysed in RIPA buffer, solubilized in the presence of 10 mM Tris/HCl pH 6.8, 2% SDS, 5% β-mercaptoethanol and resolved in 15% SDS-polyacrylamide gel for subsequent Western blot analysis with the following primary antibodies: rabbit anti-AGC1/SLC25A12 (cat. ab246329), rabbit anti-AGC2/SLC25A13 (cat. ab249408), rabbit anti-histone H3 (acetyl K9) (cat. ab32129), and rabbit anti-histone H3 (acetyl K27) (cat. ab4729) purchased from Abcam (Waltham, MA, USA); mouse anti-GAPDH (cat. 437000) and mouse anti-citrate synthase (cat. MA5-17264) from ThermoFisher; rabbit anti-PARP1 (cat. 9532), rabbit anti-Caspase3 (cat. 9661), rabbit anti-tri-methyl-histone H3 (Lys4) (cat. 9751), rabbit anti-tri-methyl-histone H3 (Lys9) (cat. 13969) from Cell Signaling (Danvers, MA, USA). The anti-rabbit (cat. 7074) and anti-mouse (cat. 7074) IgG HRP-conjugated secondary antibodies from Cell Signaling were used. Labeled proteins were visualized using Immobilon Western ECL Substrate (cat. WBKLS0500, Merck) and detected with a ChemiDocTM MP imaging system. Densitometric analysis was performed using Image Lab TM Touch software (BioRad Laboratories).

**Respiratory chain complexes analysis.**

Mitochondrial respiratory chain complex and citrate synthase activities were measured as previously described [30]. The enzymatic reactions of NADH-ubiquinone oxidoreductase (complex I), succinate dehydrogenase (complex II), Complex II + III (succinate cytochrome *c* reductase), cytochrome *c* oxidase (complex IV), and ATP synthase (complex V) were measured in permeabilized NPs (1x10^6^ cells per assay) using a Cary 50 spectrophotometer (Agilent Technologies) for 2 min at a controlled temperature with a linear slope in the presence or absence of specific inhibitors when present.

**Quantitative RT-PCR.**

Total RNA from NPs was extracted in triplicate using TRIzol Reagent (cat. 15596026, ThermoFisher), and transcribed into cDNA using the PrimeScript RT Master Mix Kit (cat. RR036A-1, Takara). Quantitative RT-PCR analysis was performed using a QuantStudioTM 3 System (Applied Biosystem) with the following predesigned Taqman gene expression assays (ThermoFisher): *MPC1* (ID Hs00211484_m1), *MPC2* (ID: Hs00967250_m1), *IDH1* (ID: Hs04966975_g1), *IDH2* (ID: Hs00953879_m1), *IDH3A* (ID:Hs00194253_m1), *IDH3B* (ID: Hs00199382_m1), *IDH3G* (ID: Hs00188065_m1), *GOT1* (ID: Hs00157798_m1), *GOT2* (ID: Hs00905827_g1), *CIC* (ID: Hs01105608_g1), *DIC* (ID: Hs00201730_m1), *OGC* (ID: Hs01087948_g1), *UCP2* (ID: Hs01075227_m1), *UCP5* (ID: Hs01073976_m1), *MDH1* (ID: Hs00936497_g1), *MDH2* (ID: Hs00938918_m1), *GLS1* (ID: Hs01014020_m1), *GLS2* (ID: Hs00998733_m1), *GC1* (ID: Hs00368705_m1), *GC2* (ID: Hs01017349_m1) and *PPIA* (ID: Hs04194521_s1) used as reference gene. qRT-PCR of *PPARGC1A*, *PPARGC1B* and *TFAM*, compared to TATA box binding protein *TBP* as housekeeping gene was performed as previously detailed [35], using the following forward (FW) and reverse (REV) primers: PPARGC1A FW, GCCAAACCAACAACTTTATCTCTTC; PPARGC1A REV, CACACTTAAGGTGCGTTCAATAGTC; PPARGC1B FW, AGTCAACGGCCTTGTGTTAAG; PPARGC1B REV, ACAACTTCGGCTCTGAGACTG; TFAM FW, AATCTGTCTGACTCTGAAAAGGA; TFAM REV, TGTGCGACGTAGAAGATCCT; TBP FW, TGCACAGGAGCCAAGAGTGAA; TBP REV, CACATCACAGCTCCCCACCA. For mtDNA relative quantification, 5 ng of DNA extracted in triplicates from NPs was amplified using primers recognizing the mitochondrial gene *MT-ND5* (FW: ATCCTTCTTGCTCATCAGTTG, REV: GGCTATTTGTTGTGGGTCTC) and the nuclear reference gene Tubulin gamma 1 *TUBG1* (FW: CCCTGGCTACATGAACAATG, REV: GTAGCCGGTCATGAGGAAGT). qRT-PCR was then performed as previously detailed [35]. Data are presented as mean ± SD and analysis was performed using the comparative 2^-ΔΔCt method. Statistical significance was assessed using a one-way ANOVA with Tukey’s test.
